# Successful application of genome sequencing in a diagnostic setting: 1007 index cases from a clinically heterogeneous cohort

**DOI:** 10.1038/s41431-020-00713-9

**Published:** 2020-08-28

**Authors:** Aida M. Bertoli-Avella, Christian Beetz, Najim Ameziane, Maria Eugenia Rocha, Pilar Guatibonza, Catarina Pereira, Maria Calvo, Natalia Herrera-Ordonez, Monica Segura-Castel, Dan Diego-Alvarez, Michal Zawada, Krishna K. Kandaswamy, Martin Werber, Omid Paknia, Susan Zielske, Dimitar Ugrinovski, Gitte Warnack, Kapil Kampe, Marius-Ionuț Iurașcu, Claudia Cozma, Florian Vogel, Amal Alhashem, Jozef Hertecant, Aisha M. Al-Shamsi, Abdulrahman Faiz Alswaid, Wafaa Eyaid, Fuad Al Mutairi, Ahmed Alfares, Mohammed A. Albalwi, Majid Alfadhel, Nouriya Abbas Al-Sannaa, Willie Reardon, Yasemin Alanay, Arndt Rolfs, Peter Bauer

**Affiliations:** 1 CENTOGENE AG, Rostock, Germany; 2 Division of Pediatric Genetics, Department of Pediatrics, Prince Sultan Military Medical City, Riyadh, Saudi Arabia; 3 Department of Pediatrics, Tawam Hospital, Al-Ain, United Arab Emirates; 4 Division of Genetics, Department of Pediatrics, King Saud bin Abdulaziz University for Health Sciences, King Abdulaziz Medical City, Riyadh, Saudi Arabia; 5 Division of Genetics, Department of Pediatrics, King Abdullah Specialized Children Hospital, King Abdulaziz Medical City, MNGHA, Riyadh, Saudi Arabia; 6 King Abdullah International Medical Research Center (KAIMRC), King Saud bin Abdulaziz University for Health Sciences, MNGHA, Riyadh, Saudi Arabia; 7 Department of Pediatrics, College of Medicine, Qassim University, Qassim, Saudi Arabia; 8 Pathology and Laboratory Medicine, King Abdulaziz Medical City, Riyadh, Saudi Arabia; 9 John Hopkins Aramco Health Care, Pediatric Services, Dhahran, Saudi Arabia; 10 Clinical Genetics, Children’s Health Ireland (CHI), Crumlin, Ireland; 11 Pediatric Genetics Division, Department of Pediatrics, School of Medicine, Acibadem Mehmet Ali Aydinlar University, Istanbul, Turkey; 12 University of Rostock, Rostock, Germany

**Keywords:** Genetics research, Genomics

## Abstract

Despite clear technical superiority of genome sequencing (GS) over other diagnostic methods such as exome sequencing (ES), few studies are available regarding the advantages of its clinical application. We analyzed 1007 consecutive index cases for whom GS was performed in a diagnostic setting over a 2-year period. We reported pathogenic and likely pathogenic (P/LP) variants that explain the patients’ phenotype in 212 of the 1007 cases (21.1%). In 245 additional cases (24.3%), a variant of unknown significance (VUS) related to the phenotype was reported. We especially investigated patients which had had ES with no genetic diagnosis (*n* = 358). For this group, GS diagnostic yield was 14.5% (52 patients with P/LP out of 358). GS should be especially indicated for ES-negative cases since up to 29.6% of them  could benefit from GS testing (14.5% with P/LP, *n* = 52 and 15.1% with VUS, *n* = 54). Genetic diagnoses in most of the ES-negative/GS-positive cases were determined by technical superiority of GS, i.e., access to noncoding regions and more uniform coverage. Importantly, we reported 79 noncoding variants, of which, 41 variants were classified as P/LP. Interpretation of noncoding variants remains challenging, and in many cases, complementary methods based on direct enzyme assessment, biomarker testing and RNA analysis are needed for variant classification and diagnosis. We present the largest cohort of patients with GS performed in a clinical setting to date. The results of this study should direct the decision for GS as standard second-line, or even first-line stand-alone test.

## Introduction

Stepwise genetic testing is a common approach to achieve molecular diagnosis in patients with suspected genetic diseases. However, such a testing approach is often time consuming, costly, and stressful for the families, particularly when no immediate diagnosis is found. Consequently, after years of testing many patients remain undiagnosed. At present, common testing strategies include chromosomal microarray analysis (CMA), single gene analysis, massive parallel sequencing of panels of selected genes, and exome and genome sequencing (ES and GS).

Despite the successful application of ES in the clinical practice [1–4], there are known technical limitations such as incomplete coverage of exonic regions, lack of coverage of deep intronic and regulatory regions, introduction of PCR artefacts during library preparations and uneven sequence depth [5–7]. Clinical GS has been introduced as the most comprehensive genetic test enabling the detection of single nucleotide variants (SNV) and small insertion–deletions (indels) [8], copy number variation (CNV) [9, 10] and structural variants (SV) [11, 12]. GS is technically superior to ES, given the relatively uniform depth of the sequencing across the genome, the coverage of exonic and intronic regions, negligible PCR bias, and trustable identification of CNVs and SVs, which is comparable or even superior to CMA [13].

Few studies evaluating the usefulness of GS in the clinical practice are available [14, 15]. In a recent meta-analysis on diagnostic utility of CMA, ES, and GS that included 20,068 children, only 374 cases with GS were available [16]. The diagnostic yield of GS in this sample set was 41%. For ES, the diagnostic yield was 36% (based on 9169 patients). They were both significantly higher than the standard of care testing (CMA, 11% in 11,429 samples). Clark et al. concluded that ES and GS should be considered as the first-line genetic tests in children with suspected genetic diseases [16].

Our study presents the results of 1007 consecutive GS cases with a broad spectrum of clinical presentations and geographic origin, which represents the largest group of patients with GS reported to date. Aiming to aid clinical decisions in the diagnostic strategy, we investigated the diagnostic utility of GS in this large dataset, and particularly analyzed the reasons defining superiority of GS over ES.

## Materials and methods

### Genome sequencing (GS)

DNA was extracted from EDTA blood or from dried blood spots on filter cards (CentoCard^®^) using standard, spin column-based methods. Genomic DNA was fragmented by sonication and Illumina adapters were ligated to generated fragments for subsequent sequencing on the HiSeqX platform (Illumina) to yield an average coverage depth of at least 30×. An average coverage of 41× was obtained in this sample set.

### Bioinformatics GS pipeline

Raw sequence data analysis, including base calling, de-multiplexing, alignment to the hg19 human reference genome (Genome Reference Consortium GRCh37), and variant calling, was performed using the HiSeq Analysis Software v2.0 pipelines (Illumina, Inc., San Diego, CA). The short reads were aligned to the GRCh37 (hg19) build of the human reference genome using Isaac aligner algorithm [17]. Variant calling was performed on the alignment files SNVs, and indels using Starling Small Variant Caller [17]. Canvas [10] and Manta [18] were used for detecting SVs and CNVs. Variants were annotated using SnpEff [19] and in-house ad hoc bioinformatics tools [1]. A collection of in silico prediction tools were applied to evaluate the conservation and possible effect of the detected variants: FATHMM, PROVEAN, SIFT, PolyPhen2-HDIV, ada_score, rf_score, MutationTaster,VEST3, LRT, MutationAssessor, MetaSVM, MetaLR, MCAP, REVEL, MutPred, CADD, DANN, GERP++NR, GERP++RS, phyloP100way_vertebrate, phyloP20way_mammalian, phastCons100way_vertebrate, and phastCons20way_mammalian. All the values were fetched from the dbNSFP database [20].

### Variant evaluation and classification

Cases were evaluated by trained scientists and human geneticists. Selection of the variants for reporting was done taking into account the compatibility with the suspected phenotype and expected disease mechanism. The clinical information was ‘translated’ into human phenotype ontology (HPO) terms, registered in our data repository and applied for each individual analysis during variant filtration and prioritization. Variant nomenclature followed standard recommendations [21]. Exon numbering was done as per the respective reference sequence indicated in Table 2 and Supplementary information Tables 2 and 3.

Pathogenic and likely pathogenic (LP) variants have been submitted to ClinVar with accession numbers from SCV001426469 to SCV001426653 (https://www.ncbi.nlm.nih.gov/clinvar/submitters/279559/).

### Confirmation of variants by an additional method

Confirmation of selected variants was done by Sanger sequencing, MLPA, qPCR, or CMA.

A description of these methods can be found in Supplementary information.

Selected candidate variants were classified according to the published ACMG guidelines as pathogenic (P), likely pathogenic (LP) and variant of unknown significance (VUS), with respect to a disease and inheritance pattern [22, 23]. P/LP variants are considered disease—causing for the specific condition and mode of inheritance.

Interpretation of the findings was performed in the clinical context, with reports being issued as: (a) positive, for P/LP variant(s) explaining the phenotype(s), (b) potential, for variants formally classified as VUS but with high evidence and compatible phenotype, (c) unclear, for VUS compatible with the clinical phenotype (at least partially), (d) negative, no relevant variant identified.

### Patients

Consent for GS testing and genetic diagnoses was given by patients, parents, or referring physicians. Data from 1007 diverse, consecutive index cases where GS was requested, were collected from a period of 2 years (2017–2018). The index cases were evaluated in a routine diagnostic setting. For the purpose of this research, all cases were individually reviewed based on all provided documentation and reports. Data regarding country of origin, family history, consanguinity, disease onset, motive of referral, and previous genetic testing were extracted from our database and individually curated.

## Results

Demographics from the included 1007 patients are shown in Table 1.

**Table 1:** General characteristics of the cohort of 1007 consecutive GS cases.

Features	Cohort of all GS case (*n* = 1007)
Age at onset	Range: prenatal–59 years
Prenatal	165 (16%)
0–5 years old	386 (38%)
6–16 years old	50 (5%)
Older than 16 years old	24 (2%)
Unknown	382 (38%)
Age at testing	
Prenatal	11 (1%)
0–5 years old	501 (50%)
6–16 years old	329 (33%)
Older than 16 years old	138 (14%)
Unknown	28 (9%)
Family history	
Positive	357 (35%)
Negative	382 (38%)
Unknown	268 (27%)
Consanguinity	
Yes	513 (51%)
No	338 (34%)
Unknown	156 (15%)
Geographical origin	
North America	39 (4%)
Latin America	37 (4%)
Europe	114 (11%)
Middle East–North Africa	785 (78%)
Asia–Australia	32 (3%)
Previous genetic testing	
ES	358 (36%)
CMA	165 (16%)
Panel	80 (8%)
Other	235 (23%)
Total number of tests	838
GS test design	
Solo	476 (47%)
Trio	437 (44%)
Other	94 (9%)

Disease onset varied from early in the prenatal period to cases with late clinical presentation (Table 1). For most cases, an early disease onset was reported (prenatal—5 years, 64%). At the time of testing, half of the cases were young children (<5 years old, *n* = 501). The proportion of patients that reported positive or negative family history was similar (35% and 37%, respectively), while 51% of the cases reported consanguinity (*n* = 513). The latter is consistent with the geographical origin of the cases given that 78% (*n* = 785) came from the Middle Eastern region where intra-familiar marriages are more common (Table 1).

We considered previous genetic testing performed either by us or elsewhere. In 36% of the cases, ES had been performed previously (*n* = 358). In 23%, ‘other’ types of genetic tests had been performed (*n* = 235) and included karyotyping, Fragile X testing, MLPA analysis, methylation, and repeat expansion analysis.

To assess the delay of genetic diagnosis, we evaluated the time elapsed between age at disease onset and age at testing/diagnosis in 130 positive cases from whom the data were available. On average, the patients waited 5 years to receive a genetic diagnosis. Forty percent of the patients received a diagnosis 1–5 years after disease onset (*n* = 52), with 15% (20 patients) receiving a diagnosis after 10 years of disease onset (range 0–50 years). Sadly, in some cases the diagnosis was reached post-mortem.

GS testing was mainly requested as two modalities, namely ‘solo’ (476 singleton, 47%) or ‘trio’ (index and parents, *n* = 437, 44%). In 9% of the cases, ‘other’ design was used (*n* = 94, e.g., healthy parents of deceased affected child, two affected siblings plus parents).

Based on provided HPOs, the motive of referral largely varied among cases. Yet, there was preponderance of neurological diseases (abnormality of the nervous system: reported in 792 patients), followed by abnormality of the head and neck (511 patients), abnormality of the skeletal system (485 patients) and abnormality of the musculature (471 patients). Less frequently reported HPOs were abnormality of the endocrine system (67 patients) and neoplasm (36 patients). The number of HPOs ranged from 1 to 52 with an average of 11 terms per patient.

### Established genetic diagnosis based on the detection of P/LP variants among 1007 GS index patients

**Fig. 1:**
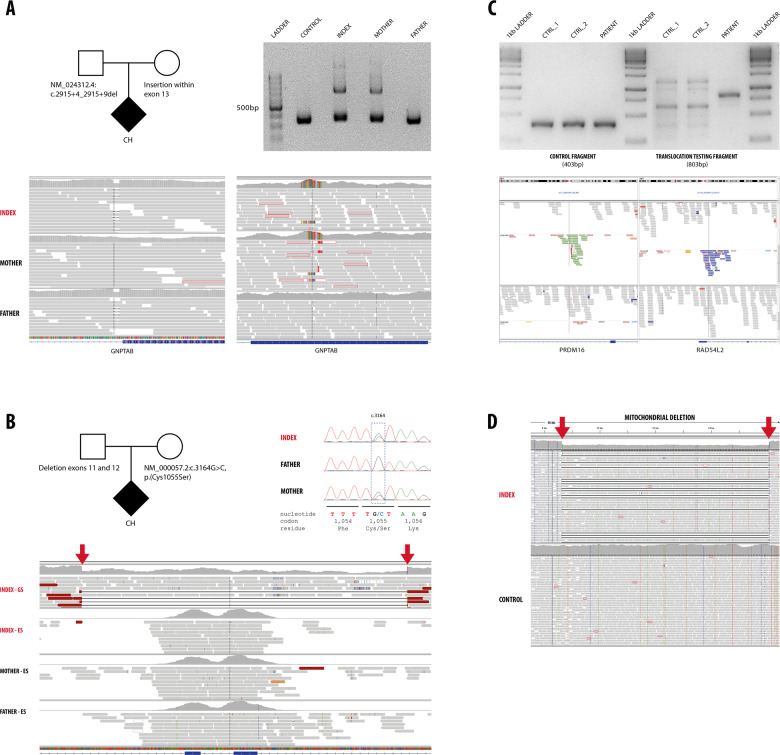
**a** The index case presented with intrauterine growth retardation, microcephaly, dysmorphism, with clinical suspicion of a lysosomal disease. GS detected a heterozygous intronic deletion (NM_024312.4(*GNPTAB)*:c.2915+4_2915+9del, inherited from the father) and a maternally inherited heterozygous insertion within exon 13 of the *GNPTAB* gene (mucolipidosis type II). Corresponding IGV images are shown with the small intronic deletion (left) and large exonic insertion (right). The exonic insertion was confirmed by PCR and gel electrophoresis, index and mother presented a larger band corresponding to the allele with the insertion. **b** The index patient presented with neurodevelopmental delay, microcephaly, abnormal skin pigmentation, reticular rash and photophobia. GS identified a heterozygous missense variant (NM_000057.2(*BLM*):c.3164G>C, p.(Cys1055Ser), Sanger traces are shown) and a heterozygous deletion encompassing exons 11 and 12 of the *BLM* gene (indicated by red arrows, IGV). Note comparation in IGV of ES and GS data (only index, upper lane) in the corresponding region of the *BLM* gene. **c** The index patient presented with neurodevelopmental delay, short stature, facial dysmorphism (hypertelorism, low set ears) and cardiovascular malformation (coarctation of the aorta and persistent ductus arteriosus). A structural variant was detected by GS: a balanced translocation between chromosomes 1p (left panel) and 3p (right panel) with break points definitions at chr1:3,300,737 and chr3:51,573,020. The break points are likely affecting the *PRDM16* and *RAD54L2* genes which have been implicated in cardiomyopathy and neurodevelopmental delay. Upper panel: Additional confirmation was performed, by agarose gel electrophoresis (1.5%) of PCR amplified products of control fragment and translocation testing fragment. Control fragment shows amplification corresponding to size 403 bp for two controls samples (CTRL_1, CTRL_2) and patient. Translocation testing fragment shows amplification corresponding to size 803 bp only for patient sample. **d** Index case presented with failure to thrive, fatigue, metabolic acidosis, polyuria, polydipsia. GS did not detect any abnormality in the nuclear DNA. A large heteroplasmic deletion was detected in the mitochondrial DNA (chrM:8637–16072, indicated by red arrows) confirming the diagnosis of mitochondrial deletion syndrome.

From 212 cases with P/LP variants, the majority (*n* = 186, 88%) had a SNV detected. Five of these cases presented a concomitant CNV. Three of these patients had biallelic variants with heterozygous SNV and CNV affecting the same gene (in trans) (examples are shown in Fig. 1a, b). The two additional cases appeared with a combination of SNV and CNV affecting different genes and providing a dual diagnosis (neuronal ceroid lipofuscinosis type 3 plus C1q deficiency and COL1A1-related osteogenesis imperfecta plus Pitt-Hopkins-like syndrome type 2).

Thirty-one cases had a CNV/SV detected (22 deletions, 8 insertions, 1 translocation, Fig. 1c). CNVs varied from deletions or duplication of single exons, to large alterations consistent with microdeletion/microduplication syndromes (1q43–q44 deletion syndrome, 2q24.3 microduplication-associated epileptic spasm), or affecting the mitochondrial genome (Fig. 1d). Recurrent events occurring in at least two patients were detected in two genes, *SMN1* and *GLA* (two novel intronic insertions in *GLA)*. The causative effect of intronic *GLA* insertions was confirmed in both patients (male and female) by pathologic enzymatic and biomarker results in blood confirming the diagnosis of Fabry disease [24].

Furthermore, 41  P/LP noncoding variants were reported (37 SNV and 4 CNV, Table 2). They included variants located in the proximity of exons, but also in regulatory and deep intronic regions. Classification of these variants is challenging given the unknown effect on the protein function. To aid classification, we performed biochemistry testing in several cases (Table 2).

**Table 2:** Intronic pathogenic and likely pathogenic variants detected by GS.

Variant	Variant coordinates	Zygosity	Class	OMIM phenotype	Supporting evidence
**NM_000339.2(*SLC12A3*):c.1670–191C>T**	**NC_000016.9:g.56917770C>T**	Hom	P	Gitelman syndrome, AR (OMIM^®^: 263800)	PMID: 19668106 and 21051746. Activating cryptic splicing site
NM_021008.3(*DEAF1*):c.997+2_997+3del	NC_000011.9:g.680960_680961del	Hom	LP	Dyskinesia, seizures, and intellectual developmental disorder, AR (OMIM^®^: 617171)	PMID: 26048982 and 24668509. Affecting splicing
**NM_014714.3(*IFT140*):c.1525–1G>A**	**NC_000016.9:g.1621536C>T**	CH with exonic P sub	LP	Mainzer-Saldino syndrome, AR (OMIM^®^: 266920)	Affecting canonical splicing site
NM_005859.4(*PURA*):c.-12_25del	NC_000005.9:g.139493755_139493791del	Het	LP	Intellectual disability type 31, AD (OMIM^®^: 616158)	37 bp deletion affecting the translation initiation codon
**NM_000548.3(*TSC2*):c.848+281C>T**	**NC_000016.9:g.2107460C>T**	Het	P	Tuberous sclerosis type 2, AD (OMIM^®^: 613254)	PMID: 10533066 and 11068191. Activating a splice donor site
**NM_001163435.2(*TBCK*):c.1170+1G>A**	**NC_000004.11:g.107163626C>T**	Hom	LP	Infantile hypotonia with psychomotor retardation and characteristic facies type 3, AR (OMIM^®^: 616900)	Affecting canonical splicing site, one additional patient in CentoMD^®^
NM_000277.1(*PAH*):c.1066–11G>A	NC_000012.11:g.103237568C>T	Hom	P	Phenylketonuria, AR (OMIM^®^: 261600)	PMID: 25596310, 25087612, 23500595 and two additional patients in CentoMD^®^
NM_020751.2(*COG6*):c.1167–24A>G	NC_000013.10:g.40273614A>G	Hom	P	Congenital disorder of glycosylation type 3, AR (OMIM^®^: 614576, OMIM^®^: 615328)	PMID: 23606727, two additional patients in CentoMD^®^
**NM_020680.3(*SCYL1*):c.1386+1G>T**	**NC_000011.9:g.65302854G>T**	Hom	LP	Spinocerebellar ataxia type 21, AR (OMIM^®^: 616719)	Affecting canonical splicing site
NM_032801.4(*JAM3*):c.612+1G>T	NC_000011.9:g.134014890G>T	Hom	P	Hemorrhagic destruction of the brain, subependymal calcification, and congenital cataracts, AR (OMIM^®^: 613730)	Affecting canonical splicing site
NM_001172086.1(*PEX2*):c.-17–2A>G	NC_000008.10:g.77896433T>C	Hom	LP	Peroxisome biogenesis disorder type 5A/B, AR (OMIM^®^: 614866, OMIM^®^: 614867)	Affecting canonical splicing site
NM_181789.2(*GLDN*):c.1028–2A>T	NC_000015.9:g.51693788A>T	Hom	LP	Lethal congenital contracture syndrome type 11, AR (OMIM^®^: 617194)	Affecting canonical splicing site
NM_001323544.1(*GALNS*):c.441–862C>T	NC_000016.9:g.88905035G>A	CH with exonic P sub	LP	Mucopolysaccharidosis IVA, AR (OMIM^®^: 253000)	Predicted to create a new splicing site. Pathological enzyme levels (galactosamine-6-sulfate sulfatase)
NM_001127511.2(*APC*):c.-190G>A	NC_000005.9:g.112043225G>A	Het	P	Familial adenomatous polyposis, AD (OMIM^®^: 175100)	PMID: 27087319
**NM_024757.4(*EHMT1*):c.21+1_21+5del**	**NC_000009.11:g.140513502_140513506del**	Het	LP	Kleefstra syndrome, AD (OMIM^®^: 610253)	Affecting canonical splicing site, de novo
NM_000518.4(*HBB*):c.315+1G>A	NC_000011.9:g.5247806CT	Het	P	Beta-thalassemia, AD (OMIM^®^: 613985)	Affecting canonical splicing site, PMID: 7151176 and 26193974
NM_001029835.2(*CCM2*):c.535+1G>C	NC_000007.13:g.45104246G>C	Het	LP	Cerebral cavernous malformations type 2, AD (OMIM^®^: 603284)	Affecting canonical splicing site
**NM_019096.4(*GTPBP2*):c.1236+1G>A**	**NC_000006.11:g.43591669C>T**	Hom	LP	Jaberi-Elahi syndrome, AR (OMIM^®^: 617988)	Affecting canonical splicing site
NM_001171.5(*ABCC6*):c.2248–2_2248–1del	NC_000016.9:g.16272823_16272824del	CH with 2 exonic VUS sub	P	Pseudoxanthoma elasticum, AR (OMIM^®^: 264800)	Affecting canonical splicing site, PMID: 15459974
NM_000528.3(*MAN2B1*):c.2356–2A>G	NC_000019.9:g.12760032T>C	Hom	LP	Alpha-mannosidosis, AR (OMIM^®^: 248500)	Affecting canonical splicing site
NM_000169.2(GLA):c.1000–72_100–58delins(2400)	NC_000023.10:g.100653145_ 100653159delins(2400)	Hem	P	Fabry disease, XL (OMIM^®^: 301500)	Pathological lyso-Gb3 biomarker, pathological alpha galactosidase quantification
**NM_007055.3(*POLR3A*):c.1771–7C>G**	**NC_000010.10:g.79769440G>C**	Hom	P	Hypomyelinating leukodystrophy type 7, AR (OMIM^®^: 607694)	PMID: 28459997
NM_014362.3(*HIBCH*):c.386–1G>C	NC_000002.11:g.191152365C>G	Het	LP	3-hydroxyisobutryl-CoA hydrolase deficiency, AR (OMIM^®^: 250620)	Affecting canonical splicing site
NM_000521.3(*HEXB*):c.1082+5G>A	NC_000005.9:g.74011520G>A	Hom	P	Sandhoff disease, AR (OMIM^®^: 268800)	PMID: 22848519
NM_000463.2(*UGT1A1*):c.-3275T>G	NC_000002.11:g.234665659T>G	Hom	LP	Genetic susceptibility to Gilbert syndrome, AR (OMIM^®^: 143500)	PMID: 11906189, 29137095, 20057336
NM_000463.2(*UGT1A1*):c.-41_-40dup	NC_000002.11:g.234668893_234668894dup	Hom	P	Genetic susceptibility to Gilbert syndrome, AR (OMIM^®^: 143500)	PMID: 7565971, 9653159
NM_000169.2(*GLA*):c.762_763ins(300)	NC_000023.10:g.100653811_100653812ins(300)	Het	P	Fabry disease, XL (OMIM^®^: 301500)	Pathological lyso-Gb3 biomarker
NM_000124.3(*ERCC6*):c.543+1G>T	NC_000010.10:g.50738765C>A	Hom	LP	Cockayne syndrome type B, AR (OMIM^®^: 133540)	Affecting canonical splicing site
NM_000271.4(*NPC1*):c.2795+56C>T	NC_000018.9:g.21119719G>A	Hom	LP	Niemann-Pick disease type C1, AR (OMIM^®^: 257220)	Pathological lyso-SM-509 biomarker
NM_020451.2(*SELENON*):c.872+1G>A	NC_000001.10:g.26135642G>T	Hom	LP	Muscular dystrophy rigid spine type 1, AR (OMIM^®^: 602771)	Affecting canonical splicing site
NM_000518.4*(HBB)*:c.316–106C>G	NC_000011.9:g.5247062G>C	Het	P	Beta-thalassemia (minor), AD (OMIM®: 613985)	PMID: 19657842, 23425204
NM_005629.3*(SLC6A8)*:c.1255–35_1272del	NC_000023.10:g.152959550_152959602del	Het	LP	Cerebral creatine deficiency syndrome type 1, XL (OMIM®: 300352)	Deletion affecting the canonical splicing site
NM_018010.3(*IFT57*):c.585+3A>G	NC_000003.11:g.107932775T>C	Hom	LP	Orofaciodigital syndrome type XVIII, AR (OMIM^®^: 617927)	Predicted to disrupt the highly conserved donor splice site of exon 4
NM_022370.3(*ROBO3*):c.767–1G>A	NC_000011.9:g.124740060G > A	CH with exonic LP del	LP	Gaze palsy, familial horizontal, with progressive scoliosis, type 1, AR (OMIM^®^: 607313)	Affecting canonical splicing site
**NM_001282281.1(*PYCR1*):c.621+1G>A**	**NC_000017.10:g.79892801C>T**	CH with exonic P dup	P	Cutis laxa type IIB/IIIB, AR (OMIM^®^: 612940 / 614438)	Affecting canonical splicing site
**NM_000152.3(*GAA*):c.-32–13T>G**	**NC_000017.10:g.78078341T>G**	Hom	P	Glycogen storage disease type II, AR (OMIM^®^: 232300)	PMID: 7881425, co-segregation in affected sibling, alpha-1,4-glucosidase pathologically decreased
NM_015910.6(*WDPCP*):c.253+2T>C	NC_000002.11:g.63713674A>G	Het-Het with intronic VUS	LP	Bardet-Biedl syndrome type 15, AR (OMIM®: 615992)	Affecting canonical splicing site
NM_000117.2(*EMD*):c.82+1G>A	NC_000023.10:g.153607927G>A	Hem	LP	Emery-Dreifuss muscular dystrophy type 1, XLR (OMIM^®^: 310300)	Affecting canonical splicing site
**NM_001079537.1(*TRAPPC6B*):c.149+2T>A**	**NC_000014.8:g.39628685A>T**	Hom	LP	Neurodevelopmental disorder with microcephaly, epilepsy, and brain atrophy, AR (OMIM^®^: 617862)	Affecting canonical splicing site
NM_001286704.1(*UFM1*):c.-273_-271del	NC_000013.10:g.38923902_38923904del	Hom	P	Hypomyelinating leukodystrophy type 14, AR (OMIM^®^: 617899)	PMID: 28931644, co-segregating in affected sibling
NM_001849.3(*COL6A2*):c.1817–3C>G	NC_000021.8:g.47545376C>G	Hom	P	Bethlem myopathy type 1 / Ullrich congenital muscular dystrophy type 1, AR (OMIM^®^: 158810 / 254090)	PMID: 15689448 and two additional patients in CentoMD^®^

Cases which had ES before are indicated in bold.

*Sub* substitution, *Del* deletion, *Dup* duplication, *Ins* insertion, *Hom* homozygous, *Het* heterozygous, *Hem* hemizygous, *Part of Het-Het* heterozygous variants with unknown phase, *CH* compound het, proven *trans* phase of alleles.

Supporting evidence for classification of noncoding variants as P/LP was obtained via parallel biochemical testing, additional unrelated patients in CentoMD^®^, previous functional work/publications, or specific variant location (e.g., canonical splicing sites, Table 2). Furthermore, RNA studies were performed to assess the putative effect of an intronic duplication in intron 7 of the *RARS2* gene (heterozygous g.88253327–88253723dup). Indeed, specific cDNA PCRs in the area from exon 6–8 confirmed the presence of an additional product of 300 bps in the cDNA of this patient, compared with the normal product of 180 bps (Supplementary information Fig. 1).

A detailed list of 233  P/LP variants reported in this study is presented in Supplementary information Table 2.

Thirty-eight additional noncoding variants were reported as VUS (Supplementary information Table 3), illustrating the difficulties in interpretation of noncoding variation. These variants were mainly novel or very rare, located in deep intronic areas, with insufficient or no functional evidence available (Supplementary information Table 3).

Most cases with positive genetic diagnosis presented autosomal recessive diseases (AR, *n* = 125, 59.0%), followed by autosomal dominant (AD, *n* = 67, 31.5%), X-linked inheritance (XL, *n* = 19, 8.5%), and mitochondrial (1 case, 0.5%). Thirty-one cases presented de novo variants; 27 of them located in AD genes, three in XL genes, and in one case, in AR gene (*LGI4*).

### GS diagnostic yield

For evaluation of GS diagnostic yield, we first considered the complete cohort of 1007 index cases. In addition, we evaluated the subgroup of cases where no previous ES testing was done but GS was used as first-line diagnostic test (*n* = 649).

**Fig. 2:**
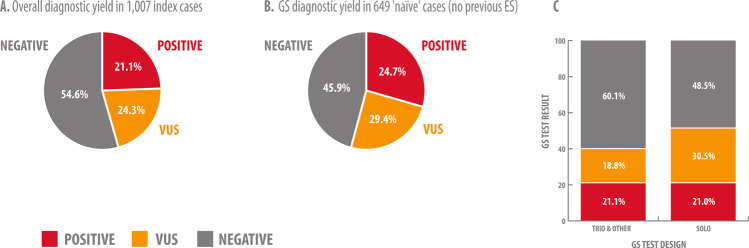
GS diagnostic yield in 1007 index cases. **a** Overall diagnostic yield in 1007 index cases. **b** GS diagnostic yield in 649 ‘naive’ cases (no previous ES). **c** Variant classification and diagnostic yield are influenced by GS test design (trio/other vs. solo). Singleton GS testing resulted in increased number of VUS reported due to difficulties phasing the alleles and recognition of de novo status, which indirectly affects diagnostic yield.

We identified P/LP variants that explain the patients’ phenotype in 212 of the 1007 cases (21.1%, Fig. 2a). GS diagnostic yield in 649 cases with no previous ES performed was higher: 24.7% (160 cases, Fig. 2b). In this ‘naive’ cohort, there was no particular enrichment for cases with positive family history or consanguinity.

We were also interested in the group of cases that had previous ES testing with negative/inconclusive results to identify factors leading to GS-based diagnosis in these ‘complex’ cases (*n* = 358, continued below). A flowchart showing GS results and previous ES testing is presented in Supplementary information Fig. 2.

### Parameters influencing diagnostic yield of GS

We assessed whether diagnostic yield is influenced by any of the parameters suggesting a genetic etiology of the disease, such as positive family history, consanguinity, or early disease onset. We also evaluated other related factors such as previous genetic testing or recentness of the GS testing. Somewhat surprisingly, neither age at disease onset, family history, nor parental consanguinity had significant impact on diagnostic yield in this cohort (Table 3). Regarding recentness, there is a trend of GS yield to increase with time/year of testing (*p* = 0.089), with most recently performed GS cases having a diagnostic yield of 23.3%. The only significant factor influencing diagnostic yield was related to pre-testing; in cases which had already received another test with no diagnosis, yield was 20.3%, while in cases for which no previous testing was mentioned (‘naïve case’), it rose to 24.1% (*p* = 0.030) (Table 3).

**Table 3:** Impact of selected parameters on diagnostic yield of GS.

Parameter	Categories	Positive cases	Negative cases	Diagnostic yield [%]	Odds ratio	*p* value (two-sided Fisher’s exact test)^a^
Age at onset	After 4 weeks of age	93	326	22.2	1.12	*0.535*
	Before 4 weeks of age	41	166	19.8		
Family history	Positive	76	281	21.3	1.16	*0.355*
	Negative	70	312	18.3		
Consanguinity	No	74	264	21.9	1.08	*0.606*
	Yes	104	409	20.3		
Recentness	Amongst the most recent 503 cases	117	386	23.3	1.23	*0.089*
	Amongst the first 504 cases	95	409	18.8		
Previous testing	No previous testing mentioned	114	359	24.1	1.19	***0.03***
	At least one previous test	98	436	20.3		

^a^The significance of the differences in diagnostic yield was tested by applying the two-sided Fisher’s exact test to the corresponding 2 × 2 matrices (positive/negative × category A/category B).

Bold values indicate statistical significance *p*-value.

In a more detailed analysis, we next focused on the type of pre-tests performed. There were 300 cases for which a single previous test was mentioned. Diagnostic yield did not significantly differ from that in naïve cases when CMA, panel sequencing or ‘other’ tests had been performed. However, when the previous test had been ES, yield significantly dropped to 14.9% (*p* = 0.021, Supplementary information Table 1).

Test design referred to the number of individuals tested per family and varied from solo testing (only index) to trio testing (index and parents) and other modalities. We observed a clear enrichment of VUS reported in the group of ‘singleton’ testing (30.5%, *n* = 145) vs. trio and other testing (18.8%, *n* = 100), indicating the positive impact of trio testing on variant classification and final diagnosis (Fig. 1c).

### Factors leading to a positive GS after negative or inconclusive ES

From 1007 cases who underwent GS, 358 reported that ES had previously been performed with no conclusive diagnosis.

In 52 cases (14.5%) of those with ES history, a P/LP variant was identified by GS which provided a genetic diagnosis. In 54 additional cases (15.1%), a VUS was reported which could be relevant for the phenotype of the index. Thus, up to 29.6% of the cases with no diagnosis established by ES, could benefit from GS testing.

To investigate the factors leading to a positive diagnosis in cases with previously negative ES, we examined the group of 273 patients that had ES performed by us (33 with positive GS diagnosis). Factors influencing GS success were grouped in two main categories: (1) time interval between tests (*n* = 12, 35%), (2) technical superiority of GS, e.g., coverage of exonic and intronic regions (*n* = 11, 33%) and detection of CNV (*n* = 10, 32%).

#### Time-interval between tests

In 12 cases, diagnosis was reached by GS, and retrospective analysis showed that the variant had been detected by ES; however, there was no scientific evidence at the time of ES testing showing a genotype–phenotype association. Importantly, in three cases the diagnosis was reached before published evidence was available, based on internal data analysis of patients in our data repository. These cases presented with variants in *NKX6*-2 [25], *PRUNE1* [26], and *UGDH* [27].

#### GS technical superiority

Eleven cases were diagnosed only via GS given that the causal variants were not detected by ES due to the lack of coverage of deep intronic areas, (NM_000548.3(*TSC2*):c.848+281C>T and NM_000339.2(*SLC12A3*):c.1670–191C>T) or poorly covered exonic variants (e.g., heterozygous NM_172362.2(*KCNH1*):c.1070G>A). This variant was detected as occurring de novo, in a case with neurodevelopmental delay (NDD), regression, intellectual disability, muscular hypertonia, spasticity, and sensorineural hearing impairment. Inspection of original sequencing data revealed that the variant was poorly covered and not detected by ES at the time. Other examples included small heterozygous deletions detected with very low quality in ES data.

Ten patients received a positive diagnosis due to CNVs detected by GS. Nine presented deletions (seven homozygous, two heterozygous). One patient presented a homozygous copy gain (4 copies), comprising a complete exon and not detected by CMA.

We also examined the group of 12 cases with positive GS diagnosis which previously tested negative by ES performed elsewhere (no raw data or specific testing details were available). Successful GS diagnosis was mainly based on detection of intronic variants (four cases), small indels (four cases), and detection of coding variants in genes that were considered as ‘diagnostic’ based on observations from our own database (*NUDT2* [28], *GTPBP2* [29], and *NKX6*-*2* [25]), allowing genetic diagnosis before that published evidence became available.

In addition, 165 cases underwent CMA testing before GS. Twenty-one received a positive diagnosis with GS, predominantly with the detection of SNVs. However, CNVs were also detected in two cases. The first case had a normal CMA result; using GS, we detected a homozygous duplication in the *INSR* gene (spanning 63 kb and affecting exon 2) below the detection limit of CMA. Variants that affect function of the *INSR* gene cause Donohue syndrome, which matched the clinical suspicion in the patient.

The second case is from a consanguineous family with two similarly affected siblings with neonatal seizures, infantile spasms, and severe NDD. CMA performed elsewhere in one sibling detected a de novo duplication in 2q. Since this was detected as a de novo event, the duplication was considered to not explain the phenotype of the affected sibling, and a GS was requested with the suspicion of a second genetic disease in the family. Using GS in both affected children, a pathogenic copy gain (3×) in chromosome 2q, including *SCN2A* gene, was detected. Other duplications in this gene are known as causal of infantile spasms. The copy gain was detected in both affected siblings and it was absent from the parents, suggesting a germinal mosaicism (maternity and paternity were confirmed).

## Discussion

We present the results of GS in 1007 consecutive index cases, from a highly heterogeneous cohort of patients. A genetic diagnosis was established in 212 cases with a diagnostic yield of 21.1%. This raised to 24.7% in the group of patients who had GS as first-line genetic test.

Consanguinity, age at onset and family history were similar in the group of cases with positive diagnosis, compared to the total cohort. None of these features had impact on diagnostic yield (Table 3). Similarly, Clark et al. [16] did not detect any impact of consanguinity on diagnostic yield in their ES/GS meta-analysis. They found increased odds of diagnosis using trios compared to singletons. In our study, singleton testing led to higher number of VUS reported, for example, in cases where phasing of the alleles or de novo status remained unknown due to the lack of parental DNA. While diagnostic yield remained similar in both groups, trio testing allowed the exclusion of variants otherwise classified as VUS. Currently, the most commonly recommended ES/GS testing design is as trio (index and parents). Other designs with inclusion of multiple affected relatives (e.g., in AD diseases) or affected male patients and maternal grandfather (for suspected XL disorders) can be considered according to the suspected mode of inheritance.

When considering recentness, we observed an increasing trend in diagnostic yield over time, with recently tested cases having the highest yield. This is in line with previous reports that showed the same trend [16]. GS is a relatively new technology, with ongoing development of tools, expanding databases for analysis, and improving interpretation of the genetic findings. The growth of databases, allowing access to allele frequencies and genotype–phenotype associations, as well as the increase of the genetic knowledge are expected to boost diagnosis utility of GS in the coming years.

We show a significant effect of previously performed ES in GS diagnostic yield. In reported cohorts with ES requested, genetic diagnosis was reached in ~30% of the patients [1, 16], with only the most complex cases remaining undiagnosed and suggesting further GS testing. Until recently, clinicians were confronted with the dilemma of indicating panel sequencing, i.e., restricting the search of variants to certain number of genes, or having a broader, genomic approach using ES. Today the dilemma resides in whether ES or GS should be indicated as first-line test. The higher cost and often less broadly accessible GS often favor an ES testing strategy.

Patients that remain undiagnosed with ES should at least be offered GS. As shown here, up to 29.6% of these patients could benefit from subsequent GS testing. The main disadvantages of the stepwise approach are longer (waiting) time to diagnosis and higher costs in such cases, supporting the rationale for using GS as single first-tier genetic testing.

Delayed diagnosis or receiving the wrong diagnosis may lead to the use of inappropriate and potentially harmful treatment and other inadequate clinical decisions [30]. In this study, the average diagnosis time was 5 years, with 40% of the patients having waited 1–5 years before receiving a diagnosis. Similarly, Molster et al. recently reported that around 50% of adult patients with rare diseases waited one year or more to be diagnosed, with almost a third waiting five or more years [31].

Two main features distinguish our study from previous reports on the diagnostic yield of GS. Here we studied a (1) large and (2) clinically heterogeneous cohort, while previous studies focused on specific groups of patients/diseases and had small sample sizes. For example, Stavropoulos et al. reported a diagnostic yield of 34% in 100 pediatric cases [15], while Farnaes et al. reported 43% diagnostic yield in a small cohort of 42 acutely ill infants [32]. Through the combined ES/GS meta-regression analysis, Clark et al. showed a significant inverse relationship between study size and reported diagnostic yield [16]. Sample size and pre-selection or diversity of cohorts are relevant parameters when evaluating reports on diagnostic utility, with smaller, more focused studies reporting higher diagnostic rates.

Clinical information is of great value when performing GS evaluation for diagnostic purposes; this might explain the higher diagnostic yield in studies that focused in a specific group of diseases. Referring physicians must be aware about the importance of the phenotypic information for proper evaluation of the GS and the molecular diagnosis of the patients.

In our study, up to 29.6% of the unsolved ES cases could benefit from GS testing. One of the features of GS that offers an advantage over ES is the homogenous coverage of exonic and noncoding regions. We indeed identified cases with P/LP variants affecting deep intronic and other noncoding regions, which would be difficult/impossible to achieve with ES. Cases with exonic variants that are difficult to detect by ES were also diagnosed in our cohort. Similarly, Belkadi et al. showed a better detection of exonic variants via GS with higher detection of true exonic variants and lower detection of false positive ones [7].

The uniform and robust coverage and depth is also important for accurate CNV/SV detection. Previous work has shown that GS-based CNV detection can be successfully employed to examine gene dosage, and to serve as a diagnostic tool [13, 33]. Among current cases, we detected deletions and insertions, not limited to exons, but also located in intronic areas. Notably, some of the CNVs were below the detection limit of CMA, e.g., within exons.

Most recent ES designs and current analysis pipelines provide better coverage and trustable CNVs detection. This might result in a smaller number of exome-negative/genome-positive cases than reported here. The advantage of GS resides in the detection of smaller CNVs given the high number of clipped reads supporting CNV calls, the covering of both coding and noncoding areas and the detection of SVs.

An apparently balanced translocation was detected in one of our cases. Analysis of GS data not only allowed the detection of the translocation but the precise mapping of the breakpoints for a better interpretation of the functional consequences (Fig. 1c). Recently, Schluth-Bolard et al. used GS to study 55 patients with intellectual disability caused by known translocations and complex chromosomal re-arrangements, concluding that GS is a valid strategy to study SVs in a clinical setting [12].

With the increasing quality of the ES during the last years, the interest of GS is shifting to the discovery of clinically relevant noncoding variation. GS provides the tools to discover and asses these variants. Recently, Cassini et al. reported a deep intronic variant in *IGHMBP2* shown to lead to nonsense mediated decay via activation of a cryptic splicing site in a patient with Charcot-Marie-Tooth [34]. As part of a more complex mechanism, Kragesteen et al. described a deletion in the first noncoding exon of *H2AFY* that leads to abnormal expression of *Pitx1* and Liebenberg syndrome [35].

In the current study, we reported 79 P/LP/VUS noncoding variants; from these, 41 were classified as P/LP. However, interpretation of noncoding variants remains challenging, and in many cases, complementary methods, for example via metabolomics, are needed to understand their functional impact. For several variants, we provided additional evidence favoring pathogenicity based on direct enzyme assessment and/or our internally developed biomarkers, allowing accurate variant classification and interpretation. Recently, other methods such as RNA-seq, have been applied in combination with ES/GS to improve diagnostic yield [36]. Thus, alternative functional methods should be considered to obtain evidence of the functional impact of the variants.

In 12 patients, the P/LP variant detected by GS was present in ES data. Reanalysis or re-evaluation of good quality NGS data or variants detected has been shown to be of great diagnostic value for both ES and GS [37–39]. Based on these results, we recommend to also consider ES reanalysis before conducting GS. However, due to its superior quality of data, GS data are of greater value when repeating evaluation and analysis until a diagnosis is reached or as soon as the phenotypes evolves [37]. GS data also provide a solid basis for research aiming to identify novel genes associated to rare diseases.

In conclusion, we present the largest cohort of patients with GS performed on a clinical setting to date. Detection of noncoding variants, improved detection of exonic variants and CNV/SV contributed to the diagnosis of many cases. GS was especially valuable in patients for whom previous ES had resulted negative. Our results highlight the strength of GS as the most comprehensive genetic test and should encourage the decision of using GS as the first-line test in complex undiagnosed patients. Updated guidelines regarding GS application in the clinical practice are urgently needed; in times of genomic medicine, GS should become the ‘standard of care’ genetic test.

## Supplementary information

Supplementary information

## References

[1] Trujillano D, Bertoli-Avella AM, Kumar Kandaswamy K, Weiss ME, Koster J, Marais A (2017). Clinical exome sequencing: results from 2819 samples reflecting 1000 families. Eur J Hum Genet.

[2] Retterer K, Juusola J, Cho MT, Vitazka P, Millan F, Gibellini F (2016). Clinical application of whole-exome sequencing across clinical indications. Genet Med.

[3] Vissers L, van Nimwegen KJM, Schieving JH, Kamsteeg EJ, Kleefstra T, Yntema HG (2017). A clinical utility study of exome sequencing versus conventional genetic testing in pediatric neurology. Genet Med.

[4] Farwell KD, Shahmirzadi L, El-Khechen D, Powis Z, Chao EC, Tippin Davis B (2015). Enhanced utility of family-centered diagnostic exome sequencing with inheritance model-based analysis: results from 500 unselected families with undiagnosed genetic conditions. Genet Med.

[5] Meienberg J, Bruggmann R, Oexle K, Matyas G (2016). Clinical sequencing: is WGS the better WES?. Hum Genet.

[6] Lelieveld SH, Spielmann M, Mundlos S, Veltman JA, Gilissen C (2015). Comparison of exome and genome sequencing technologies for the complete capture of protein-coding regions. Hum Mutat.

[7] Belkadi A, Bolze A, Itan Y, Cobat A, Vincent QB, Antipenko A (2015). Whole-genome sequencing is more powerful than whole-exome sequencing for detecting exome variants. Proc Natl Acad Sci USA.

[8] Van der Auwera GA, Carneiro MO, Hartl C, Poplin R, Del Angel G, Levy-Moonshine A (2013). From FastQ data to high confidence variant calls: the Genome Analysis Toolkit best practices pipeline. Curr Protoc Bioinform.

[9] Abyzov A, Urban AE, Snyder M, Gerstein M (2011). CNVnator: an approach to discover, genotype, and characterize typical and atypical CNVs from family and population genome sequencing. Genome Res.

[10] Roller E, Ivakhno S, Lee S, Royce T, Tanner S (2016). Canvas: versatile and scalable detection of copy number variants. Bioinformatics.

[11] Sudmant PH, Rausch T, Gardner EJ, Handsaker RE, Abyzov A, Huddleston J (2015). An integrated map of structural variation in 2,504 human genomes. Nature.

[12] Schluth-Bolard C, Diguet F, Chatron N, Rollat-Farnier PA, Bardel C, Afenjar A (2019). Whole genome paired-end sequencing elucidates functional and phenotypic consequences of balanced chromosomal rearrangement in patients with developmental disorders. J Med Genet.

[13] Gross AM, Ajay SS, Rajan V, Brown C, Bluske K, Burns NJ (2019). Copy-number variants in clinical genome sequencing: deployment and interpretation for rare and undiagnosed disease. Genet Med.

[14] Petrikin JE, Cakici JA, Clark MM, Willig LK, Sweeney NM, Farrow EG (2018). The NSIGHT1-randomized controlled trial: rapid whole-genome sequencing for accelerated etiologic diagnosis in critically ill infants. NPJ Genom Med.

[15] Stavropoulos DJ, Merico D, Jobling R, Bowdin S, Monfared N, Thiruvahindrapuram B (2016). Whole genome sequencing expands diagnostic utility and improves clinical management in pediatric medicine. NPJ Genom Med.

[16] Clark MM, Stark Z, Farnaes L, Tan TY, White SM, Dimmock D (2018). Meta-analysis of the diagnostic and clinical utility of genome and exome sequencing and chromosomal microarray in children with suspected genetic diseases. NPJ Genom Med.

[17] Raczy C, Petrovski R, Saunders CT, Chorny I, Kruglyak S, Margulies EH (2013). Isaac: ultra-fast whole-genome secondary analysis on Illumina sequencing platforms. Bioinformatics.

[18] Chen X, Schulz-Trieglaff O, Shaw R, Barnes B, Schlesinger F, Kallberg M (2016). Manta: rapid detection of structural variants and indels for germline and cancer sequencing applications. Bioinformatics.

[19] Cingolani P, Platts A, Wang le L, Coon M, Nguyen T, Wang L (2012). A program for annotating and predicting the effects of single nucleotide polymorphisms, SnpEff: SNPs in the genome of *Drosophila melanogaster* strain w1118; iso-2; iso-3. Fly.

[20] Liu X, Wu C, Li C, Boerwinkle E (2016). dbNSFP v3.0: a one-stop database of functional predictions and annotations for human nonsynonymous and splice-site SNVs. Hum Mutat.

[21] den Dunnen JT, Dalgleish R, Maglott DR, Hart RK, Greenblatt MS, McGowan-Jordan J (2016). HGVS recommendations for the description of sequence variants: 2016 update. Hum Mutat.

[22] South ST, Lee C, Lamb AN, Higgins AW, Kearney HM, Working Group for the American College of Medical G. (2013). ACMG standards and guidelines for constitutional cytogenomic microarray analysis, including postnatal and prenatal applications: revision 2013. Genet Med.

[23] Richards S, Aziz N, Bale S, Bick D, Das S, Gastier-Foster J (2015). Standards and guidelines for the interpretation of sequence variants: a joint consensus recommendation of the American College of Medical Genetics and Genomics and the Association for Molecular Pathology. Genet Med.

[24] Lukas J, Scalia S, Eichler S, Pockrandt AM, Dehn N, Cozma C (2016). Functional and clinical consequences of novel alpha-galactosidase A mutations in Fabry disease. Hum Mutat.

[25] Baldi C, Bertoli-Avella AM, Al-Sannaa N, Alfadhel M, Al-Thihli K, Alameer S (2018). Expanding the clinical and genetic spectra of NKX6-2-related disorder. Clin Genet.

[26] Zollo M, Ahmed M, Ferrucci V, Salpietro V, Asadzadeh F, Carotenuto M (2017). PRUNE is crucial for normal brain development and mutated in microcephaly with neurodevelopmental impairment. Brain.

[27] Hengel H, Bosso-Lefevre C, Grady G, Szenker-Ravi E, Li H, Pierce S (2020). Loss-of-function mutations in UDP-glucose 6-dehydrogenase cause recessive developmental epileptic encephalopathy. Nat Commun.

[28] Yavuz H, Bertoli-Avella AM, Alfadhel M, Al-Sannaa N, Kandaswamy KK, Al-Tuwaijri W (2018). A founder nonsense variant in NUDT2 causes a recessive neurodevelopmental disorder in Saudi Arab children. Clin Genet.

[29] Bertoli-Avella AM, Garcia-Aznar JM, Brandau O, Al-Hakami F, Yuksel Z, Marais A (2018). Biallelic inactivating variants in the GTPBP2 gene cause a neurodevelopmental disorder with severe intellectual disability. Eur J Hum Genet.

[30] Gahl WA, Mulvihill JJ, Toro C, Markello TC, Wise AL, Ramoni RB (2016). The NIH undiagnosed diseases program and network: applications to modern medicine. Mol Genet Metab.

[31] Molster C, Urwin D, Di Pietro L, Fookes M, Petrie D, van der Laan S (2016). Survey of healthcare experiences of Australian adults living with rare diseases. Orphanet J Rare Dis.

[32] Farnaes L, Hildreth A, Sweeney NM, Clark MM, Chowdhury S, Nahas S (2018). Rapid whole-genome sequencing decreases infant morbidity and cost of hospitalization. NPJ Genom Med.

[33] Ellingford JM, Campbell C, Barton S, Bhaskar S, Gupta S, Taylor RL (2017). Validation of copy number variation analysis for next-generation sequencing diagnostics. Eur J Hum Genet.

[34] Cassini TA, Duncan L, Rives LC, Newman JH, Phillips JA, Koziura ME (2019). Whole genome sequencing reveals novel IGHMBP2 variant leading to unique cryptic splice-site and Charcot-Marie-Tooth phenotype with early onset symptoms. Mol Genet Genom Med.

[35] Kragesteen BK, Brancati F, Digilio MC, Mundlos S, Spielmann M (2019). H2AFY promoter deletion causes PITX1 endoactivation and Liebenberg syndrome. J Med Genet.

[36] Deelen P, van Dam S, Herkert JC, Karjalainen JM, Brugge H, Abbott KM (2019). Improving the diagnostic yield of exome- sequencing by predicting gene-phenotype associations using large-scale gene expression analysis. Nat Commun.

[37] Costain G, Jobling R, Walker S, Reuter MS, Snell M, Bowdin S (2018). Periodic reanalysis of whole-genome sequencing data enhances the diagnostic advantage over standard clinical genetic testing. Eur J Hum Genet.

[38] Need AC, Shashi V, Schoch K, Petrovski S, Goldstein DB (2017). The importance of dynamic re-analysis in diagnostic whole exome sequencing. J Med Genet.

[39] Wright CF, McRae JF, Clayton S, Gallone G, Aitken S, FitzGerald TW (2018). Making new genetic diagnoses with old data: iterative reanalysis and reporting from genome-wide data in 1,133 families with developmental disorders. Genet Med.

