# Supplementary material for: Successful application of genome sequencing in a diagnostic setting: 1007 index cases from a clinically heterogeneous cohort
Source: Eur J Hum Genet. 2020 Aug 28;29(1):141–53. doi: 10.1038/s41431-020-00713-9 (PMC7852664; doi:10.1038/s41431-020-00713-9)
Supplement: Supplementary file 1 — Supplementary information [file 41431_2020_713_MOESM1_ESM.docx]

**SUPPLEMENTARY INFORMATION**

**Successful application of genome sequencing in a diagnostic setting: 1,007 index cases from a clinically heterogeneous cohort**

**Supplementary methods**

Exome sequencing

In a subset of the samples, ES had been performed by us as described previously ^1^. During the last six years we used several kits and platforms: Ion torrent- AmpliSeqExome, Illumina kit - Nextera rapid capture exome v 1.2, Agilent kit S07604514, SureSelect Human All Exon V6 and WESTwist. Size of target region varied from 33 to 60 Mb from CCDS, RefSeq and Gencode database with average depth of ≥100X, with 97 to 98% of targeted regions covered at ≥20X.

Confirmation of variants by an additional method

Confirmation of selected variants was done by Sanger sequencing, MLPA, qPCR or CMA. Sanger sequencing from both sides on a 3730xl sequencer (Thermo Fisher Scientific, Waltham, MA). Genome-wide copy number variation + SNP analysis was performed using CytoScan® 750K Array and CytoScan® HD Array according to manufacturer`s protocols (Thermo Fisher Scientific Inc.). Results were analyzed with the Chromosome Analysis Suite software (ChAS, Affymetrix, Inc., Santa Clara, CA). Analysis thresholds were as follows: Het deletions with a minimum of 25 markers and/or a size >50kb; homozygous deletions with at least 5 aberrant markers and a size >1kb; duplications >200kb; regions with absence of heterozygosity (AOH) >3Mb. MLPA® analyses were performed with commercially available kits according to manufacturer`s instructions (MRC-Holland, Amsterdam, The Netherlands). MLPA reactions were run in ABI 3730xl / 3130xl DNA Analyzers (Applied Biosystems). To confirm copy number variants when no commercially available MLPA kit was available, we performed quantitative PCR assays (qPCR). When possible, in-house designs targeting 2-3 exons within the copy number variant and 1-2 additional fragments outside the alteration were used. Products were run in a LightCycler 480 II (Roche). Segregation of the variant(s) was evaluated in available family members.

RNA/cDNA analysis

Blood was collected in PAXgene™ Blood RNA Tubes (PreAnalytiX GmbH, Hombrechtikon, Switzerland), RNA was extracted with PAXgene™ Blood RNA Kit (PreAnalytiX GmbH, Hombrechtikon, Switzerland) and cDNA was generated with Invitrogen SuperScript™ IV First-Strand Synthesis System (Thermo Fisher Scientific, Vilnius, Lithuania). Primers hybridizing to intron-spanning exons were designed to specifically amplify cDNA. PCR amplicons from index and control samples were run on 1% agarose gel to assess potential aberrant splicing.

**Supplementary figures**

Figure 1. Aberrant splicing caused by a heterozygous intronic insertion in *RARS2,* detected in a patient with an additional pathogenic heterozygous variant in the same gene. cDNA based PCR detected a larger, abnormal band of 300 bps, compared to control cDNAs that showed only the expected 180 bps band. This finding supports the pathogenicity of the intronic insertion detected by GS.
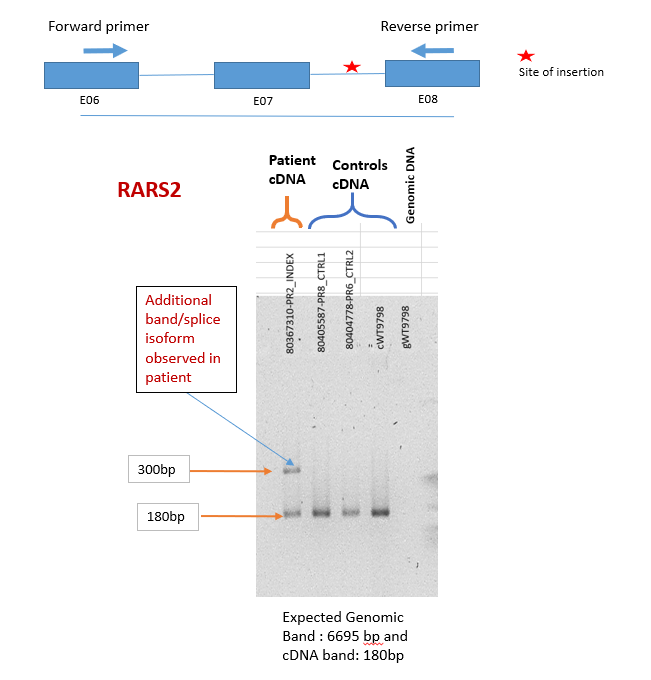


Figure 2. Flowchart showing GS results and previous ES testing. P/PL indicate pathogenic and likely pathogenic variants. VUS: variant of unknown significance. Causes identified as leading to a positive (P/LP) GS based diagnosis after a negative ES are summarized based on 33 positive cases with ES performed at CENTOGENE.


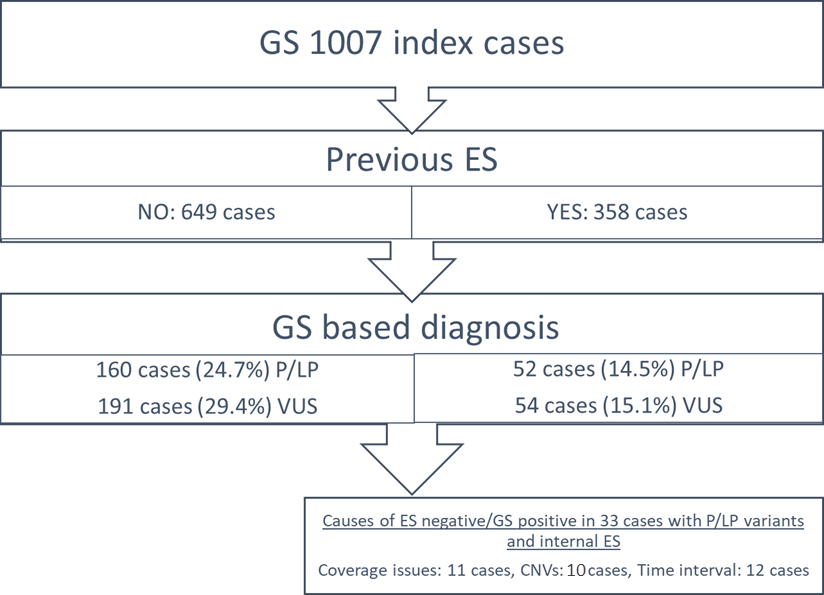


**Supplementary tables**

**Table 1.** Impact of distinct single previous tests on diagnostic yield of GS

| Previous test | Categories | Positive cases | Negative cases | Diagnostic yield [%] | Odds ratio | p-value (two-sided Fisher’s exact test) * |
| --- | --- | --- | --- | --- | --- | --- |
| *Chromosomal microarray (CMA)* | Previously only CMA | 7 | 11 | 38.9 | 1.61 | *0.166* |
|  | No previous test | 114 | 359 | 24.1 |  |  |
| *Panel*  *sequencing (PS)* | Previously only PS | 5 | 33 | 13.2 | 0.55 | *0.162* |
|  | No previous test | 114 | 359 | 24.1 |  |  |
| *Exome sequencing (ES)* | Previously only ES | 21 | 120 | 14.9 | 0.62 | ***0.021*** |
|  | No previous test | 114 | 359 | 24.1 |  |  |
| *Other* | Previous only “other” test | 25 | 78 | 24.3 | 1.01 | *1.000* |
|  | No previous test | 114 | 359 | 24.1 |  |  |

***** The significance of the differences in diagnostic yield was tested by applying the two-sided Fisher’s exact Test to the corresponding 2 x 2 matrices (positive/negative x category A/category B)

**Table 2.** Pathogenic and likely pathogenic variants reported in this cohort with patient’s clinical information (HPOs). Variant nomenclature is according to Genome Reference Consortium GRCh37 (hg19).

| Ref.Seq. and nt change | Protein change | Zyg. | Class | Disease | MOI | HPOs |
| --- | --- | --- | --- | --- | --- | --- |
| NM_001282628.1(ARSE):c.1818G>A | p.(Trp606*) | Hem | P | Chondrodysplasia punctata | XL | Coarse facial features,Kyphoscoliosis |
| NM_024757.4(EHMT1):c.21+1_21+5del | p.? | Het | LP | Kleefstra syndrome | AD | Micropenis,Delayed speech and language development,Hyperactivity,Intellectual disability,Muscular hypotonia,Global developmental delay,Motor delay,Abnormal corpus callosum morphology,Abnormal facial shape,Attention deficit hyperactivity disorder |
| NM_020680.3(SCYL1):c.1386+1G>T | p.? | Hom | LP | Spinocerebellar ataxia type 21 | AR | Microcephaly,Intellectual disability,Hepatic failure,Hepatosplenomegaly,Failure to thrive,Elevated hepatic transaminase |
| NM_000153.3(GALC):c.334A>G | p.(Thr112Ala) | Hom | P | Krabbe disease | AR | Muscular hypotonia,Global developmental delay,Hyperreflexia |
| NM_021222.2(PRUNE1):c.88G>A | p.(Asp30Asn) | Hom | LP | PRUNE1-related disorders | AR | Spasticity,Motor delay,Brain atrophy |
| NM_001042537.1(SLC9A6):c.1299_1300del | p.(Phe434Hisfs*8) | Hem | LP | Christianson type syndromic mental retardation | XL | Visual impairment,Ptosis,Ophthalmoplegia,Seizures,Global developmental delay,Cerebellar atrophy,Muscle weakness,Lissencephaly,Abnormal cortical gyration,Abnormal circulating cysteine concentration,Methylmalonic aciduria,Brain atrophy,Abnormal circulating creatine kinase concentration,Hypoammonemia |
| NM_001429.3(EP300):c.3067del | p.(Ile1023*) | Het | LP | Rubinstein-Taybi syndrome type 2 | AD | Oral cleft,Microcephaly,Deeply set eye,Myopia,Intellectual disability,Tremor,Growth delay,Abnormal facial shape,Dysphagia,Constipation,Narrow nasal tip |
| NM_001270447.1(ACADVL):c.494T>C | p.(Phe165Ser) | Hom | LP | VLCAD deficiency | AR | Microcephaly,Seizures,Muscular hypotonia,Global developmental delay,Generalized hypotonia,Patent ductus arteriosus,Abnormal facial shape,Generalized tonic-clonic seizures,Hypoplasia of the corpus callosum,Delayed gross motor development,Inability to walk,Difficulty standing,Severe global developmental delay,Brain atrophy,Delayed myelination,Decreased activity of 3-hydroxyacyl-CoA dehydrogenase |
| NM_001256821.1(RIT1):c.295T>G | p.(Phe99Val) | Het | P | Noonan syndrome type 8 | AD | Abnormality of the cardiovascular system,Thickened calvaria,Short stature,Reduced bone mineral density |
| NM_000169.2(GLA):c.762_763ins(300) | p.? | Het | P | Fabry disease | XL | Hypertension,Angiokeratoma corporis diffusum,Elevated serum creatinine |
| NM_001161.4(NUDT2):c.34C>T | p.(Arg12*) | Hom | P | NUDT2-associated disorder | AR | High palate,High forehead,Global developmental delay,Motor delay,Growth delay,Abnormal facial shape,Feeding difficulties,Long ear |
| NM_015915.4(ATL1):c.574C>T | p.(Leu192Phe) | Het | LP | Spastic paraplegia type 3A | AD | Spastic paraplegia |
| NM_000548.3(TSC2):c.848+281C>T | p.? | Het | P | Tuberous sclerosis type 2 | AD | Hypopigmentation of the skin,Seizures,Global developmental delay,Encephalopathy,Cerebral edema,EEG abnormality,Abnormality of the cerebral white matter,Sepsis |
| NM_000339.2(SLC12A3):c.1670-191C>T | p.? | Hom | P | Gitelman syndrome | AR | Adrenal insufficiency,Failure to thrive,Iron deficiency anemia,Renal tubular acidosis,Hypokalemia,Hypomagnesemia,Decreased circulating aldosterone level,Metabolic alkalosis |
| Chr6(DCDC2):g.24357438_24357756del | p.? | Hom | LP | Neonatal sclerosing cholangitis | AR | Cirrhosis,Patent foramen ovale,Secundum atrial septal defect,Cholestatic liver disease,Elevated hepatic transaminase,Increased urinary taurine,Prolonged neonatal jaundice,Giant cell hepatitis |
| NM_030632.1(ASXL3):c.3592_3593insGAT | p.(Leu1198*) | Het | LP | Bainbridge-Ropers syndrome | AD | Triangular mouth,Microcephaly,Prominent nasal bridge,Anteverted nares,Deeply set eye,Synophrys,Pectus excavatum,Hemangioma,Muscular hypotonia,Global developmental delay,Failure to thrive,Abnormal facial shape,Highly arched eyebrow,High, narrow palate,Decreased body weight,Spotty hypopigmentation,Delayed myelination |
| NM_001292034.2(TAB2):c.1526del | p.(Pro509Leufs*3) | Het | LP | TAB2-associated congenital heart defect | AD | Cutis laxa,Generalized hypotonia,Joint hypermobility,Mitral valve prolapse,Cardiomyopathy,Abnormal aortic valve morphology,Mitral regurgitation,Tricuspid valve prolapse |
| NM_003359.3(UGDH):c.950G>A | p.(Arg317Gln) | Hom | P | UGDH-related epileptic encephalopathy | AR | Muscular hypotonia,Global developmental delay,Abnormal facial shape |
| NM_001347465.1(C1QA):c.210delinsAA | p.(Gly71Argfs*30) | Hom | LP | C1q deficiency | AR | Visual impairment,Rod-cone dystrophy,Macular degeneration,Optic atrophy,Eczema,Atopic dermatitis,Seborrheic dermatitis,Alopecia,Macular dystrophy,Abnormal blood zinc concentration,Mixed hypo- and hyperpigmentation of the skin |
| NM_021008.3(DEAF1):c.997+2_997+3del | p.? | Hom | LP | DEAF1-associated disorder | AR | Microcephaly,Poor eye contact,Muscular hypotonia,Global developmental delay,Generalized hypotonia,Sleep disturbance,Involuntary movements,Feeding difficulties,Abnormality of movement |
| NM_177400.2(NKX6-2):c.196del | p.(Arg66Glyfs*122) | Hom | LP | Spastic ataxia type 8, with hypomyelinating leukodystrophy | AR | Nystagmus,Global developmental delay,Abnormality of the cerebral white matter,Lower limb hypertonia,Muscular hypotonia of the trunk,Abnormal circulating creatine concentration,Delayed myelination |
| NM_172362.2(KCNH1):c.1070G>A | p.(Arg357Gln) | Het | P | Zimmermann - Laband syndrome 1 | AD | Seizures,Muscular hypotonia,Global developmental delay,Abnormality of the cerebral white matter,EMG abnormality,Muscular dystrophy,Mildly elevated creatine kinase |
| NM_001127511.2(APC):c.-190G>A | p.? | Het | P | Familial adenomatous polyposis | AD | Inflammation of the large intestine,Colon cancer,Adenomatous colonic polyposis,Chronic gastritis,Rectal polyposis |
| NM_139284.2(LGI4):c.1272C>A | p.(Cys424*) | Het (CH) | LP | Neurogenic arthrogryposis multiplex congenita | AR | Abnormality of the skeletal system,Abnormality of limb bone morphology,Multiple joint contractures,Neonatal death |
| NM_139284.2(LGI4):c.263_265del | p.(Phe88del) | Het (CH) | LP | Neurogenic arthrogryposis multiplex congenita | AR | Same patient as above |
| NM_019074.3(DLL4):c.1392C>A | p.(Cys464*) | Het | LP | Adams-Oliver syndrome type 6 | AD | Aplasia cutis congenita,Short finger |
| NM_000065.3(C6):c.2049C>G | p.(Tyr683*) | Hom | LP | Complement component 6 deficiency | AR | High palate,Dolichocephaly,Short philtrum,Low-set ears,Intellectual disability,Seizures,Global developmental delay,Joint hypermobility,Joint laxity,Abnormal facial shape,Broad-based gait,Attention deficit hyperactivity disorder |
| NM_019096.4(GTPBP2):c.1527_1528del | p.(Glu509Aspfs*78) | Hom | LP | Jaberi-Elahi syndrome | AR | Microcephaly,Strabismus,Upslanted palpebral fissure,Delayed speech and language development,Poor eye contact,Global developmental delay,Motor delay,Agenesis of corpus callosum,Absent speech,Hyperreflexia,Joint hypermobility,Small for gestational age,Premature birth,Sandal gap,Abnormal facial shape,Gastroesophageal reflux,Chorea,Asthma,Delayed gross motor development,Difficulty walking,Neonatal hyperbilirubinemia,Short stature,Decreased body weight,Depressed nasal bridge,Muscular hypotonia of the trunk,Protruding tongue,Abnormal myelination,Delayed myelination |
| NM_001277115.1(DNAH11):c.6565C>T | p.(Arg2189*) | Het | LP | Primary ciliary dyskinesia 7 with or without situs inversus | AR | Parent of index with: with Polycystic kidney dysplasia |
| NM_014714.3(IFT140):c.1525-1G>A | p.? | Het (CH) | LP | Mainzer-Saldino syndrome | AR | Wide anterior fontanel,Dolichocephaly,Long face,Micrognathia,High forehead,Protruding ear,Ptosis,Long eyelashes,Nystagmus,Pectus carinatum,Narrow chest,Poor eye contact,Hyperextensible skin,Global developmental delay,Joint laxity,Failure to thrive,Calcaneovalgus deformity,Abnormal facial shape,Poor head control,Neck flexor weakness,Short stature,Severe muscular hypotonia |
| NM_014714.3(IFT140):c.1990G>A | p.(Glu664Lys) | Het (CH) | P | Mainzer-Saldino syndrome | AR | Same patient as above |
| Chr17(ALOXE3):g.8003231-8010960del | p.? | Hom | LP | Congenital ichthyosis type 3 | AR | Delayed speech and language development,Dry skin,Global developmental delay,Difficulty walking,Ichthyosis,Scaling skin on fingertip,Scaling skin |
| NM_001323544.1(GALNS):c.1492G>A | p.(Ala498Thr) | Het (CH) | P | Mucopolysaccharidosis IVA | AR | Mucopolysacchariduria |
| NM_001323544.1(GALNS):c.441-862C>T | p.? | Het (CH) | LP | Mucopolysaccharidosis IVA | AR | Same patient as above |
| NM_000303.2(PMM2):c.422G>A | p.(Arg141His) | Het (CH) | P | Congenital disorder of glycosylation, type Ia | AR | Hypogonadism,Torticollis,Strabismus,Intellectual disability,Dysarthria,Abnormal cerebellum morphology,Dystonia,Tremor,Progressive cerebellar ataxia,Intention tremor,Enlarged cisterna magna,Kyphoscoliosis |
| NM_000303.2(PMM2):c.722G>C | p.(Cys241Ser) | Het (CH) | P | Congenital disorder of glycosylation, type Ia | AR | Same patient as above |
| NM_001198868.1(CAPN1):c.1015C>T | p.(Arg339*) | Hom | LP | Spastic paraplegia type 76 | AR | Osteopenia,Gait disturbance,Obesity,Iron deficiency anemia,Back pain |
| NM_018896.4(CACNA1G):c.632T>C | p.(Leu211Pro) | Het | LP | Spinocerebellar ataxia type 42 | AD | Abnormality of the face,Abnormality of the neck,Single transverse palmar crease,Syndactyly,Muscular hypotonia,Global developmental delay,Dystonia,Hyperreflexia,Failure to thrive,Broad nail,Broad toe,Gastroesophageal reflux,Polydactyly,Feeding difficulties,Neonatal asphyxia,Abnormality of movement |
| NM_005859.4(PURA):c.-12_25del | p.? | Het | LP | Intellectual disability type 31 | AD | Macrocephaly,Intellectual disability,Muscular hypotonia,Global developmental delay,Central hypotonia |
| NM_001005360.2(DNM2):c.1856C>T | p.(Ser619Leu) | Het | P | Centronuclear myopathy type 1 | AD | Micrognathia,Cyanosis,Hand clenching,Muscular hypotonia,Motor delay,Joint stiffness,Failure to thrive,Intrauterine growth retardation,Polyhydramnios,Cardiomegaly,Patent foramen ovale,Abnormal cardiac ventricle morphology,Abnormal facial shape,Respiratory failure,Myopathy,Decreased body weight,CNS demyelination,Stridor,Abnormality of mouth shape,Abnormal brainstem MRI signal intensity,Abnormality of salivation |
| NM_014334.3(FRRS1L):c.961C>T | p.(Gln321*) | Hom | P | Early infantile epileptic encephalopathy type 37 | AR | Seizures,Muscular hypotonia,Global developmental delay,Sleep disturbance,EEG with focal spikes,Perivascular spaces |
| NM_020751.2(COG6):c.1167-24A>G | p.? | Hom | P | COG6-related disorder | AR | Behavioral abnormality,Autism,Global developmental delay,Gait disturbance,Unexplained fevers,Echolalia,Mild fetal ventriculomegaly,Abnormal social behavior,Pain,Cognitive impairment |
| NM_000518.4(HBB):c.315+1G>A | p.? | Het | P | Beta thalassemia minor | AD | Narrow forehead,Behavioral abnormality,Intellectual disability,Muscular hypotonia,Hyperreflexia,Joint laxity,Abnormal facial shape,Generalized-onset seizure,Poor head control,Inability to walk,Abnormality of calvarial morphology,High, narrow palate,Hypochromic microcytic anemia,Type D brachydactyly,Long toe,Edema of the dorsum of feet,Self-biting |
| NM_001008537.2(NEXMIF):c.791_792del | p.(Phe264Tyrfs*2) | Hem | LP | X-linked mental retardation type 98 | XLD | Same patient as above |
| NM_001178014.1(PCCB):c.944G>C | p.(Ser315Thr) | Hom | LP | Autosomal recessive propionic acidemia | AR | Seizures,Muscular hypotonia,Lethargy,Global developmental delay,Hyporeflexia,Generalized hypotonia,Hyperreflexia,Abnormality of metabolism/Homeostasis,Generalized-onset seizure,Propionicacidemia,Aciduria,Infantile spasms |
| NM_005249.4(FOXG1):c.256del | p.(Gln86Argfs*106) | Het | P | FOXG1-related syndrome | AD | Microcephaly,Strabismus,Visual impairment,Intellectual disability,Muscular hypotonia,Global developmental delay,Failure to thrive,Abnormality of the cerebral white matter,Cerebral hypomyelination |
| NM_130445.2(COL18A1):c.2969_2978del | p.(Pro990Leufs*35) | Hom | P | Knobloch syndrome type 1 | AR | Abnormal retinal morphology,Nystagmus,High myopia |
| NM_001244008.1(KIF1A):c.946C>T | p.(Arg316Trp) | Het | P | Mental retardation type 9 | AD | Oculomotor apraxia,Ataxia,Muscular hypotonia,Cognitive impairment |
| NM_005993.4(TBCD):c.1661C>T | p.(Ala554Val) | Hom | P | TBCD-related neurodevelopmental disorder | AR | Microcephaly,Delayed speech and language development,Intellectual disability,Generalized-onset seizure |
| NM_015634.3(KIF1BP):c.169G>T | p.(Glu57*) | Hom | LP | Goldberg-Shprintzen megacolon syndrome | AR | Polyhydramnios,Vomiting,Aganglionic megacolon,Abdominal distention |
| NM_001110792.1(MECP2):c.177_180dup | p.(Ser61Alafs*8) | Het | LP | Rett syndrome | XLD | Strabismus,Intellectual disability,Muscular hypotonia,Hyperreflexia,Developmental regression,Mild microcephaly |
| NM_002585.3(PBX1):c.836A>G | p.(Gln279Arg) | Het | LP | Congenital anomalies of kidney and urinary tract syndrome with or without hearing loss, abnormal ears, or developmental delay | AD | Cryptorchidism,Dolichocephaly,Narrow forehead,Prominent nose,Deeply set eye,Muscular hypotonia,Hyperreflexia,Failure to thrive,Barrel-shaped chest,Atrial septal defect,Patent ductus arteriosus,Coarctation of aorta,Fever,Abnormal facial shape,Hyperkalemia,Delayed gross motor development,Highly arched eyebrow,Achalasia,Neonatal respiratory distress,Generalized joint laxity,Recurrent upper respiratory tract infections,Pulmonary artery stenosis,Prominent metopic ridge,Esophageal stenosis,Left-to-right shunt |
| NM_014855.2(AP5Z1):c.931C>T | p.(Arg311*) | Hom | LP | Spastic paraplegia type 48 | AR | Coarse facial features,Abnormality of the skeletal system,Muscular hypotonia,Global developmental delay,Encephalopathy,Hepatosplenomegaly,Dysphagia,Kyphoscoliosis,Decreased muscle mass,Congenital blindness,Cough |
| NM_001206983.2(METTL23):c.169_172del | p.(His57Valfs*11) | Hom | P | Mental retardation type 44 | AR | Abnormality of the face,Nystagmus,Delayed speech and language development,Intellectual disability,Motor delay,Joint hypermobility,Congenital onset,Cleft lower lip |
| NM_177400.2(NKX6-2):c.487C>G | p.(Leu163Val) | Hom | P | NKX6-2 associated phenotype | AR | Metabolic acidosis,Hepatomegaly,Increased CSF lactate,Elevated hepatic transaminase,Hyperglycemia,Lactic acidosis,Hyperalaninemia,Acute encephalopathy,Abnormal circulating creatinine level |
| NM_000046.4(ARSB):c.264G>T | p.(Gln88His) | Het | P | Mucopolysaccharidosis type VI | AR | Inguinal hernia,Abnormality of the gingiva,Macrocephaly,Abnormality of the nasal bridge,Abnormality of the nervous system,Muscular hypotonia,Hepatosplenomegaly,Growth delay,Umbilical hernia,Dilated cardiomyopathy,Anemia,Abnormal facial shape,Short finger,Abnormal circulating carbohydrate concentration. Pathologic enzyme values but second variant not identified. |
| NM_000257.3(MYH7):c.5186_5188del | p.(Lys1729del) | Het | P | Laing distal myopathy | AD | Same patient as above |
| NM_183235.2(RAB27A):c.475T>C | p.(Tyr159His) | Hom | LP | Griscelli syndrome type 2 | AR | Cholestasis,Hepatic failure,Hepatosplenomegaly,Failure to thrive,Ascites,Abnormality of coagulation,Elevated hepatic transaminase |
| NM_017755.5(NSUN2):c.1020del | p.(Gly341Valfs*15) | Hom | P | Mental retardation type 5 | AR | Microcephaly,Delayed speech and language development,Intellectual disability,Muscular hypotonia,Motor delay,Short stature |
| NM_000426.3(LAMA2):c.6488del | p.(Lys2163Argfs*12) | Hom | P | Merosin-deficient congential muscular dystrophy type 1a | AR | Hyperpigmentation of the skin,Hemangioma,Muscular hypotonia,Failure to thrive,Polyhydramnios,Fasciculations,Arthrogryposis multiplex congenita,Chronic,Feeding difficulties,Cough |
| NM_001008537.2(NEXMIF):c.1882C>T | p.(Arg628*) | Het | P | Mental retardation type 98 | XL | Protruding ear,Abnormality of the eye,Behavioral abnormality,Autistic behavior,Stereotypy,Hyperactivity,Poor eye contact,Muscular hypotonia,Reduced tendon reflexes,Abnormal facial shape,Loss of speech,Epileptic spasms,Concave nasal ridge |
| NM_001321120.1(TBX4):c.1115dup | p.(Pro373Serfs*14) | Het | LP | Ischiocoxopodopatellar syndrome | AD | Proteinuria,Nephrotic syndrome,Glaucoma,Hypertension,Abnormality of the skeletal system,Gait imbalance,Scoliosis,Patellar hypoplasia,Stage 5 chronic kidney disease,Death in adolescence |
| NM_152263.2(TPM3):c.377+863_775+422del | p.? | Hom | LP | Nemaline myopathy type 1 | AR | Global developmental delay,Failure to thrive,Abnormality of the respiratory system,Abnormality of the periventricular white matter,Recurrent lower respiratory tract infections,Myopathy |
| NM_001163435.2(TBCK):c.1170+1G>A | p.? | Hom | LP | Infantile hypotonia with psyHomotor retardation and characteristic facies type 3 | AR | Muscular hypotonia,Global developmental delay,Abnormal facial shape,Feeding difficulties |
| NM_001145794.1(ANTXR2):c.652T>C | p.(Cys218Arg) | Hom | P | Hyaline fibromatosis | AR | Nephrocalcinosis,Delayed speech and language development,Spasticity,Global developmental delay,Motor delay,Limitation of joint mobility,Hepatic failure,Subcutaneous nodule,Gastroschisis,Constipation,Developmental regression,Abnormal myelination |
| NM_001354304.1(PAH):c.1066-11G>A | p.? | Hom | P | Phenylketonuria | AR | Elevated phenylalanine, developmental delay, eczema |
| NM_000050.4(ASS1):c.380G>T | p.(Arg127Leu) | Hom | P | Citrullinemia type 1 | AR | Microcephaly,Renal tubular acidosis,Decreased body weight,Elevated plasma citrulline,Medullary nephrocalcinosis,Upper limb hypertonia |
| NM_000402.3(G6PD):c.653C>T | p.(Ser218Phe) | Hem | P | Hemolytic anemia due  to G6PD deficiency | XL | Facial asymmetry,Abnormality of the pinna,Anophthalmia,Abnormality of the hand,Failure to thrive,Ventricular septal defect,Double outlet right ventricle,Abnormal facial shape,Decreased glucosephosphate isomerase activity,Neonatal onset,Absent radius,Aplasia of the ulna,Aplasia/Hypoplasia affecting the eye,External ear malformation,Unilateral radial aplasia,Oligodactyly |
| NM_005807.4(PRG4):c.3254_3260dup | p.(Val1088Glnfs*4) | Hom | LP | Camptodactylyarthropathy- coxa vara-pericarditis syndrome | AR | Hirsutism,Abnormal joint morphology,Abnormality of the cardiovascular system,Abnormal facial shape,Short stature |
| NM_138422.3(ADAT3):c.430G>A | p.(Val144Met) | Hom | P | Mental retardation type 36 | AR | Global developmental delay,Failure to thrive,Short stature,Decreased body weight |
| NM_001130987.1(DYSF):c.4076T>C | p.(Leu1359Pro) | Hom | P | Muscular dystrophy, limbgirdle type 2B | AR | Hypothyroidism,Muscle weakness,Tetraparesis,Elevated hepatic transaminase,Myoglobinuria,Skeletal muscle atrophy,Elevated serum creatine kinase,Muscular dystrophy,Skeletal muscle hypertrophy,Elevated aldolase level,Fatty replacement of skeletal muscle,Vitamin D deficiency |
| NM_177400.2(NKX6-2 ):c.608G>A | p.(Trp203*) | Hom | LP | Spastic ataxia type 8, with hypomyelinating leukodystrophy | AR | Inguinal hernia,Strabismus,Nystagmus,Intellectual disability,Seizures,Spasticity,Global developmental delay,Dystonia,Hyperreflexia,Failure to thrive,Premature birth,Abnormal facial shape,Developmental regression,Inability to walk,Scoliosis,Multiple joint contractures,Cerebral hypoplasia |
| NM_001040142.1(SCN2A):c.788C>T | p.(Ala263Val) | Het | P | Early infantile epileptic encephalopathy type 11 | AD | Delayed speech and language development,Intellectual disability,Seizures,Episodic ataxia,Focal-onset seizure |
| NM_032801.4(JAM3):c.612+1G>T | p.? | Hom | P | Hemorrhagic destruction of the brain, subependymal calcification, and congenital cataracts | AR | Hydrocephalus,Developmental cataract,Ataxia,Motor delay,Agenesis of corpus callosum,Cerebellar hypoplasia,Intrauterine growth retardation,Hypertrophic cardiomyopathy,Hypoglycemia,Abnormal facial shape,Midface retrusion |
| NM_000402.3(G6PD):c.653C>T | p.(Ser218Phe) | Hem | P | Hemolytic anemia due to glucose-6-phosphate dehydrogenase | XL | Renal hypoplasia,Polyuria,Renal cyst,Renal dysplasia,Tachycardia,Fever,Polydipsia,Palpitations,Hyperuricemia,Elevated serum creatinine,Decreased glucosephosphate isomerase activity,Stage 5 chronic kidney disease |
| NM_000094.3(COL7A1):c.6205C>T | p.(Arg2069Cys) | Het | P | Epidermolysis bullosa | AD | Micrognathia,Deeply set eye,Motor delay,Intrauterine growth retardation,Atrial septal defect,Neonatal onset,Vascular skin abnormality |
| NM_001282281.1(PYCR1):c.300_301dup | p.(Ile101Thrfs*10) | Het | LP | PYCR1-related Cutis laxa | AR | Same patient as above |
| NM_001282281.1(PYCR1):c.621+1G>A | p.? | Het | P | PYCR1-related Cutis laxa | AR | Same patient as above |
| NM_001172086.1(PEX2):c.-17-2A>G | p.? | Hom | LP | Peroxisome biogenesis disorder type 5 | AR | Abnormality of the face,Global developmental delay,Muscular hypotonia of the trunk |
| Chr1(LEPR):g.66101655_66115862del | p.? | Hom | LP | Morbid obesity due to leptin receptor deficiency | AR | Macrocephaly,Muscular hypotonia,Hepatic steatosis,Obesity,Delayed gross motor development,Hepatomegaly,Obstructive sleep apnea,Death in childhood,Increased body weight,Feeding difficulties |
| NM_000402.3(G6PD):c.653C>T | p.(Ser218Phe) | Hem | P | Glucose-6-phosphate dehydrogenase deficiency | XL | Hydrocele testis,Hypospadias,Cleft palate,Hypertension,Omphalocele,Premature birth,Patent foramen ovale,Neonatal respiratory distress,Hyponatremia,Increased serum ferritin,Decreased glucosephosphate isomerase activity |
| NM_181789.3(GLDN):c.1028-2A>T | p.? | Hom | LP | Lethal congenital contracture syndrome 11 | AR | Flexion contracture,Hydrops fetalis,Pulmonary hypoplasia,Pleural effusion |
| Chr6(DCDC2):g.24357438_24357756del | p.? | Hom | LP | Neonatal sclerosing cholangitis | AR | Splenomegaly,Hepatomegaly,Elevated hepatic transaminase,Neonatal hyperbilirubinemia,Prolonged neonatal jaundice,Abnormal urinary color,Abnormality of the ductus choledochus |
| NM_001079537.1(TRAPPC6B):c.149+2T>A | p.? | Hom | LP | Neurodevelopmental disorder with microcephaly,epilepsy, and brain atrophy | AR | Microcephaly,Delayed speech and language development,Hyperactivity,Abnormal thumb morphology,Muscular hypotonia,Global developmental delay,Motor delay,Absent speech,Obesity,Abnormal facial shape,Hypoplasia of the corpus callosum,Leukoencephalopathy,Leukodystrophy,Poor head control,Abnormal head movements,Inability to walk,EMG: chronic denervation signs,Difficulty standing,Wide intermamillary distance,Almond-shaped palpebral fissure,Muscular hypotonia of the trunk,Paraplegia,Titubation,Abnormality of movement |
| NM_001257198.1(CUL3):c.1376dup | p.(Asn459Lysfs*5) | Het | LP | Pseudohypoaldosteronism type IIE | AD | Macrocephaly,Short philtrum,Triangular face,High forehead,Low-set ears,Acne,Intellectual disability,Global developmental delay,Specific learning disability,Metabolic acidosis,Renal tubular acidosis,Abnormal facial shape,Asthma,Curly hair,High, narrow palate |
| NM_006580.3(CLDN16):c.445C>T | p.(Arg149*) | Hom | P | Renal hypomagnesemia type 3 | AR | Recurrent urinary tract infections,Duplicated collecting system,Renal cyst,Nephrocalcinosis,Hypomagnesemia,Dilatation of the renal pelvis |
| NM_006439.4(MAB21L2):c.151C>G | p.(Arg51Gly) | Het | P | Syndromic microphthalmia type 14 | AD | Eyelid coloboma,Talipes equinovarus,High, narrow palate,Short femur,Short humerus,Rhizomelia,Limb undergrowth |
| NM_001161.4(NUDT2):c.34C>T | p.(Arg12*) | Hom | P | NUTD2-associaed neurodevelopmental disorder | AR | Microcephaly,Delayed speech and language development,Muscular hypotonia,Spasticity,Motor delay,Leukodystrophy,Abnormality of the cerebral white matter,Abnormal myelination,Hypoammonemia |
| NM_152268.3(PARS2):c.283G>A | p.(Val95Ile) | Hom | P | PARS2-related disorder | AR | Wide mouth,Microcephaly,Widely spaced teeth,Hyperactivity,Intellectual disability,Absent speech,Cardiomegaly,Abnormal facial shape,Flat occiput,Mildly elevated creatine kinase |
| NM_175629.2(DNMT3A):c.2141C>G | p.(Ser714Cys) | Het | LP | Tatton-Brown-Rahman syndrome | AD | Macrocephaly,Mandibular prognathia,Hypertelorism,Abnormal eyelid morphology,Single transverse palmar crease,Muscular hypotonia,Global developmental delay,Decreased fetal movement,Polyhydramnios,Hypoglycemia,Abnormal facial shape,Dysphagia,Inverted nipples,Overlapping fingers,Flat face,Delayed myelination |
| NM_031307.3(PUS3):c.1181_1182del | p.(Ser394Cysfs*18) | Hom | LP | PUS3-related disorder | AR | Microcephaly,Visual impairment,Nystagmus,Intellectual disability,Seizures,Muscular hypotonia,Global developmental delay,Hyporeflexia,Failure to thrive,Mild hearing impairment |
| NM_022167.3(XYLT2):c.1552del | p.(Leu518Trpfs*89) | Hom | LP | XYLT2- related disorder | AR | Developmental glaucoma,Polyhydramnios,Premature birth,Ventricular septal defect,Aortic valve stenosis,Abnormal facial shape,Hepatomegaly,Abnormal circulating tyrosine concentration,Abnormal circulating Homocysteine concentration |
| NM_007055.3(POLR3A):c.1771-7C>G | p.? | Hom | P | Hypomyelinating leukodystrophy type 7 | AR | Motor delay,Generalized hypotonia,Failure to thrive,Abnormality of the basal ganglia,Developmental regression,Decreased body weight,Abnormality of the pons,Brain atrophy,Abnormal brainstem MRI signal intensity,Abnormal basal ganglia MRI signal intensity,Arachnoid cyst |
| Chr5(SMN1):g.70247660-70248318del | p.? | Hom | P | Spinal muscular atrophy | AR | Waddling gait,Inability to walk,Lumbar hyperlordosis,Myopathy,Skeletal muscle atrophy |
| NM_005458.7(GABBR2):c.2077G>T | p.(Gly693Trp) | Het | P | Early infantile epileptic encephalopathy type 59 | AD | Strabismus,Nystagmus,Delayed speech and language development,Muscular hypotonia,Global developmental delay,Motor delay,Encephalopathy,Absent speech,Vomiting,Generalized-onset seizure,Poor head control,Inability to walk,Recurrent lower respiratory tract infections,Intellectual disability, severe,Brain atrophy,Infantile spasms,Profound,Epileptic encephalopathy |
| Chr15(BLM):g.91311718-91313754del | p.? | Het | P | Bloom syndrome | AR | Microcephaly,Photophobia,Skin rash,Abnormality of skin pigmentation,Intellectual disability,Global developmental delay |
| NM_000057.2(BLM):c.3164G>C | p.(Cys1055Ser) | Het | P | Bloom syndrome | AR | Same patient as above |
| NM_020533.2(MCOLN1):c.1336G>A | p.(Val446Met) | Hom | LP | Mucolipidosis IV | AR | Coarse facial features,Delayed speech and language development,Intellectual disability,Muscular hypotonia,Global developmental delay,Motor delay,Muscle weakness,Joint laxity,Gastroesophageal reflux,Pseudobulbar signs,Recurrent bronchopulmonary infections,Corneal opacity,Abnormally lax or hyperextensible skin,Aciduria,Chronic constipation,Hyperintensity of cerebral white matter on MRI |
| NM_033419.3(PGAP3):c.850C>T | p.(His284Tyr) | Hom | LP | Mental retardation type 4 | AR | Macrocephaly,Micrognathia,Hearing impairment,Prominent nose,Long palpebral fissure,Delayed speech and language development,Intellectual disability,Muscular hypotonia,Global developmental delay,Abnormal facial shape,Short stature,Muscular hypotonia of the trunk |
| NM_000314.4(PTEN):c.529T>C | p.(Tyr177His) | Het | LP | Macrocephaly - autism syndrome | AD | Macrocephaly,Delayed speech and language development,Growth hormone excess,Cutis laxa,Hyperextensible skin,Global developmental delay,Motor delay,Large for gestational age,Abnormal facial shape,Craniofacial asymmetry,Short palpebral fissure |
| NM_017890.4(VPS13B):c.7440del | p.(Cys2480Trpfs*20) | Hom | LP | Cohen syndrome | AR | Abnormality of the midface,Delayed speech and language development,Muscular hypotonia,Global developmental delay,Abnormal facial shape,Pes valgus,High myopia |
| Chr8(MCPH1):g.6271772 -6280561del | p.? | Hom | LP | Primary microcephaly | AR | Cryptorchidism,Hypospadias,Hydrocephalus,Visual impairment,Intellectual disability,Global developmental delay,Developmental regression |
| NM_025137.3(SPG11):c.5036_5041delins256 | p.(Arg1679Ilefs*4) | Hom | LP | SPG11-related disorder | AR | Specific learning disability,Myelopathy,Difficulty walking,Motor polyneuropathy,Peripheral neuropathy,Spastic hemiparesis,Cognitive impairment |
| NM_001844.4(COL2A1):c.1457G>A | p.(Gly486Asp) | Het | LP | COL2A1-related spondyloepiphyseal dysplasia | AD | Abnormality of the skeletal system,Hip dysplasia,Spondyloepiphyseal dysplasia,Short stature |
| NM_022068.2(PIEZO2):c.8216C>T | p.(Ser2739Leu) | Het | LP | Distal arthrogryposis type 5 | AD | Myopia,Bilateral talipes equinovarus,Decreased facial expression,Bilateral camptodactyly,Distal arthrogryposis,Camptodactyly |
| NM_001029835.2(CCM2):c.535+1G>C | p.? | Het | LP | Cerebral cavernous malformations type 2 | AD | Visual loss,Ischemic stroke,Hemiplegia,Venous thrombosis,Cerebral venous thrombosis,Optic neuritis |
| NM_000091.3(COL4A3):c.305del | p.(Ser102Leufs*51) | Het (CH) | LP | Alport syndrome | AR | Renal insufficiency,Proteinuria |
| NM_000091.3(COL4A3):c.345del | p.(Pro116Leufs*37) | Het (CH) | P | Alport syndrome | AR | Same patient as above |
| NM_000088.3(COL1A1):c.2010del | p.(Gly671Alafs*95) | Het | P | COL1A1-related osteogenesis imperfecta | AD | Microcephaly,Short philtrum,Diabetes mellitus,Intellectual disability,Cerebellar atrophy,Hypoplasia of the corpus callosum,Long nose,Brain atrophy,Colpocephaly |
| Chr2(NRXN1):g.chr2:50920082_51059469del | p.? | Het | LP | Pitt-Hopkins-like syndrome type 2 | AD | Same patient as above |
| NM_019096.4(GTPBP2):c.1236+1G>A | p.? | Hom | LP | Jaberi-Elahi syndrome | AR | Muscular hypotonia,Failure to thrive,Abnormal facial shape,Neurological speech impairment,Severe sensorineural hearing impairment,Muscular hypotonia of the trunk,Severe global developmental delay |
| NM_001171.5(ABCC6):c.2248-2_2248-1del | p.? | Het | P | PseudoxantHoma elasticum, forme fustre | AD | Abnormality of vision,Abnormality of the skin |
| NM_172107.2(KCNQ2):c.1657C>T | p.(Arg553Trp) | Het | P | KCNQ2-related disorder | AD | Epileptic encephalopathy |
| NM_000271.4(NPC1):c.2795+56C>T | p.? | Hom | LP | Niemann-Pick disease type C1 | AR | Global developmental delay,Splenomegaly,Developmental regression |
| NM_001244008.1(KIF1A):c.40C>A | p.(Pro14Thr) | Het | LP | KIF1A-related disorder | AD | Intellectual disability,Spasticity,Global developmental delay,Specific learning disability,Hyperreflexia,Abnormality of the foot,Clonus,Clumsiness,Childhood onset |
| NM_018010.3(IFT57):c.585+3A>G | p.? | Hom | LP | Orofaciodigital syndrome type XVIII | AR | Ambiguous genitalia,Abnormality of the tongue,Macroglossia,Abnormality of the gingiva,Oral cleft,Dandy-Walker malformation,Nail dysplasia,Genu recurvatum,Polydactyly,Abnormality of cardiovascular system morphology,Cleft lip |
| NM_000528.3(MAN2B1):c.2356-2A>G | p.? | Hom | LP | Alpha-mannosidosis | AR | Cryptorchidism,Cleft palate,Microcephaly,Chronic otitis media,Sensorineural hearing impairment,Visual impairment,Esotropia,Delayed speech and language development,Congenital diaphragmatic hernia,Abnormality of the skeletal system,Jaundice,Eczema,Muscular hypotonia,Global developmental delay,Motor delay,Failure to thrive,Omphalocele,Laryngomalacia,Abnormality of the cardiovascular system,Ventricular septal defect,Tetralogy of Fallot,Pulmonic stenosis,Abnormal aortic valve morphology,Aortic regurgitation,Abnormal facial shape,Gastroesophageal reflux,Respiratory distress,Right aortic arch with mirror image branching,Bone pain,Sleep apnea,Cleft lip |
| NM_017799.3(TMEM260):c.1688del | p.(Thr563Lysfs*32) | Hom | LP | Structural heart defects and renal anomalies syndrome | AR | Same patient as above |
| NM_015185.2(ARHGEF9):c.354C>A | p.(Tyr118*) | Hem | LP | Early infantile epileptic encephalopathy type 8 | XL | Autism,Delayed speech and language development,Intellectual disability,Seizures,Motor delay,Maternal diabetes |
| Chr15(CLN6):g.68514779_68524790del | p.? | Hom | P | Neuronal ceroid lipofuscinosis type 6 | AR | Visual impairment,Seizures,Dysarthria,Dystonia,Myoclonus,Neurodegeneration,Developmental regression,Abnormality of the periventricular white matter,CNS demyelination,Brain atrophy |
| NM_001040142.1(SCN2A):c.2558G>A | p.(Arg853Gln) | Het | P | Early infantile epileptic encephalopathy type 11 | AD | Intellectual disability,Muscular hypotonia,Global developmental delay,Pachygyria,Abnormal cortical gyration |
| NM_000169.2(GLA):c.1000-72_1000-58delins(2400) | p.? | Hem | P | Fabry disease | XL | Proteinuria,Hypertension,Left ventricular hypertrophy,Elevated serum creatinine,Arrhythmia |
| NM_001171603.1(PREPL):c.1633C>T | p.(Gln545*) | Hom | LP | Congenital myasthenic syndrome type 22 | AR | Prominent nasal bridge,Hyperactivity,Gynecomastia,Nephrolithiasis,Growth hormone deficiency,Dry skin,Hypopigmentation of the skin,Tapered finger,Muscular hypotonia,Global developmental delay,Joint hypermobility,Hyperconvex fingernails,Abnormal facial shape,Short stature,Short finger |
| Chr2:g.164918852-167120791dup | p.? | Het | P | 2q24.3 microduplication-associated epileptic spasm | AD | Strabismus,Jaundice,Seizures,Global developmental delay,Generalized hypotonia,Muscular hypotonia of the trunk,Infantile spasms |
| NM_002317.6(LOX):c.1009C>T | p.(Arg337*) | Het | LP | Thoracic aortic aneurysm type 10 | AD | Urinary incontinence,Behavioral abnormality,Autism,Global developmental delay,Failure to thrive,Thrombocytosis,Abnormality of coagulation,Constipation,Asthma,Aortic dissection,Arthralgia,Elevated hepatic transaminase,Poor appetite,Aortic aneurysm,Lower limb muscle weakness,Fatigue,Fatigable weakness of distal limb muscles |
| NM_000521.3(HEXB):c.1082+5G>A | p.? | Hom | P | Sandhoff disease | AR | Seizures,Muscular hypotonia,Motor delay,Hyperreflexia,Premature birth,Neurodegeneration,Generalized-onset seizure,Developmental regression,Focal-onset seizure |
| NM_002292.3(LAMB2):c.4276dup | p.(Ala1426Glyfs*6) | Het | LP | Pierson syndrome | AR | Parent of index with: Renal insufficiency,Renal agenesis,Renal dysplasia,Pallor,Intrauterine growth retardation,Talipes equinovarus,Abnormal facial shape,Potter facies,Pulmonary hypoplasia,Respiratory distress,Depressed nasal bridge |
| NM_000463.2(UGT1A1):c.-3275T>G | NC_000002.11:g.234665659T>G | Hom | LP | Genetic susceptibility to Gilbert syndrome, AR (OMIM®: 143500) |  | Hyperbilirubinemia,Autism,Intelectual disability mild |
| NM_000463.2(UGT1A1):c.-41_-40dup | NC_000002.11:g.234668893_234668894dup | Hom | P | Genetic susceptibility to Gilbert syndrome, AR (OMIM®: 143500) |  | Same patient as above |
| NM_003560.3(PLA2G6):c.2222G>A | p.(Arg741Gln) | Het (CH with VUS) | P | Neurodegeneration with brain iron accumulation type 2B | AR | Delayed speech and language development,Ataxia,Developmental regression,Brain atrophy,Abnormality of movement |
| NM_001005735.1(CHEK2):c.1046G>C | p.(Gly349Ala) | Het | LP | CHEK2-related disorder | AD | Index is asymptomatic, request for cancer-related genes |
| NM_003108.3(SOX11):c.359C>T | p.(Pro120Leu) | Het | LP | Coffin-Siris syndrome | AD | Micropenis,Hypogonadism,Microcephaly,Tapered finger,Intellectual disability,Muscular hypotonia,Short stature,Decreased testicular size |
| NM_000124.3(ERCC6):c.543+1G>T | p.? | Hom | LP | Cockayne syndrome B | AR | Microcephaly,Deeply set eye,Cataract,Intellectual disability,Areflexia,Gait disturbance,Hyperreflexia,Cerebral atrophy,Gait imbalance,Caudate atrophy,Abnormal globus pallidus morphology,Involuntary movements,Generalized cerebral atrophy/hypoplasia,Nail-biting,Abnormality of the internal capsule,Stuttering,Toe walking |
| NM_005431.1(XRCC2):c.350dup | p.(Leu117Phefs*6) | Het (CH with VUS) | LP | Fanconi anemia, complementation group U | AR | Renal insufficiency,Proteinuria,Abnormality of upper lip,Epistaxis,Ptosis,Myopia,Hypertension,Urticaria,Rheumatoid arthritis,Weight loss,Abnormal facial shape,Myopathic facies,Dyspnea,Abnormality of the vasculature,Scoliosis,Systemic lupus erythematosus,Limb muscle weakness,Urticarial plaque |
| NM_018890.3(RAC1):c.198A>T | p.(Arg66Ser) | Het | LP | Mental retardation type 48 | AD | Brachycephaly,Macrocephaly,Hypertelorism,Prominent nasal bridge,Upslanted palpebral fissure,Muscular hypotonia,Global developmental delay,Abnormal facial shape,Gastroesophageal reflux,Lower limb spasticity,Frequent falls,High, narrow palate,Genu valgum,Poor fine motor coordination,Attention deficit hyperactivity disorder,CNS demyelination,Intrinsic hand muscle atrophy,Arachnoid cyst |
| NM_001267039.1(LARP7):c.448del | p.(Arg150Glufs*12) | Hom | LP | Alazami syndrome | AR | Pointed chin,Triangular face,Deeply set eye,Delayed speech and language development,Hypopigmentation of the skin,Vitiligo,Global developmental delay,Failure to thrive,Growth delay,Weight loss,Butterfly vertebrae,Chromosome breakage |
| NM_000152.3(GAA):c.266G>A | p.(Arg89His) | Het | P | Pompe disease | AR | High palate,Decreased nerve conduction velocity,Muscular hypotonia,Global developmental delay,Hyporeflexia,Motor delay,Muscle weakness,Joint hypermobility,Pes planus,Delayed gross motor development,Motor deterioration,Frequent falls,Scoliosis,Kyphoscoliosis,Myopathy,Limb-girdle muscle weakness,Gowers sign,EMG abnormality,Difficulty climbing stairs,Proximal muscle weakness,Quadriceps muscle weakness,Lower limb pain (pathologic biochemical test but second variant not identified, RNA studies ongoing). |
| NM_001110792.1(MECP2):c.952C>T | p.(Arg318Cys) | Het | P | Rett syndrome | XLD | Progressive microcephaly,Broad nasal tip,Abnormality of the optic nerve,Nystagmus,Muscular hypotonia,Global developmental delay,Plagiocephaly,Failure to thrive,Abnormal facial shape |
| Chr15(RAB27A):g.55514530-55552417dup | p.? | Hom | P | Griscelli syndrome type 2 | AR | Abnormality of skin pigmentation,Hypopigmentation of the skin,Albinism,Abnormality of the hair,Silver-gray hair,Melanin pigment aggregation in hair shafts,Prolonged neonatal jaundice,Generalized hypopigmentation,Abnormality of hair pigmentation,Abnormality of hair texture,Blue nevus |
| NM_207581.3(DUOXA2):c.95dup | p.(Leu32Phefs*78) | Het (CH with VUS) | LP | Thyroid dyshormonogenesis | AR | Behavioral abnormality,Constipation,Aganglionic megacolon,Sleep disturbance,Gastroparesis,Attention deficit hyperactivity disorder,Orthostatic tachycardia,Abnormal autonomic nervous system physiology,Fatigue,Colonic inertia,Chronic pain |
| NM_004646.3(NPHS1):c.3478C>T | p.(Arg1160*) | Het | P | Congenital nephrotic syndrome, Finnish type | AR | Parent of index with congenital nephrotic syndrome |
| Chr6(COL10A1):g.116441794-116442866del | p.? | Het | LP | Schmid-type metaphyseal chondrodysplasia | AD | Poor wound healing,Joint hypermobility,Abnormality of the abdominal organs,Aganglionic megacolon,Intestinal fistula |
| NM_172107.2(KCNQ2):c.431G>A | p.(Arg144Gln) | Het | P | Early infantile epileptic encephalopathy type 7 | AD | Hypospadias,Hydrocephalus,Low-set ears,Strabismus,Exotropia,Intellectual disability,Global developmental delay,Agenesis of corpus callosum,Absent speech,Failure to thrive,Mitral regurgitation,Hypoplasia of the corpus callosum,Intellectual disability, moderate,EEG abnormality,Sleep disturbance,Developmental regression,Poor speech,Attention deficit hyperactivity disorder,External ear malformation,Feeding difficulties in infancy,Feeding difficulties,Abnormal myelination,Abnormality of the brainstem white matter |
| NM_014727.2(KMT2B):c.1205del | p.(Pro402Hisfs*5) | Het | LP | Childhood-onset dystonia type 28 | AD | Abnormality of the dentition,Carious teeth,Ulnar claw,Dysarthria,Global developmental delay,Dystonia,Hip dysplasia,Premature birth,Bilateral talipes equinovarus,Hematemesis,Language impairment,Spastic tetraplegia,Scoliosis,Malnutrition,Cerebral palsy,Abnormality level of calcium-phosphate regulating hormone |
| NM_054027.4(ANKH):c.13C>T | p.(Pro5Ser) | Het | LP | Condrocalcinosis type 2 | AD | Same patient as above |
| NM_023035.2(CACNA1A):c.4055G>A | p.(Arg1352Gln) | Het | P | CACNA1A-associated disorder | AD | Facial grimacing,Stereotypy,Poor eye contact,Muscular hypotonia,Global developmental delay,Cerebellar atrophy,Tremor,Absent speech,Enlarged cisterna magna,Head tremor,Hand tremor,Attention deficit hyperactivity disorder,Abnormality of movement |
| NM_000091.3(COL4A3):c.1006G>T | p.(Gly336Cys) | Het | P | Alport syndrome | AD | Tinnitus,Vertigo,Microscopic hematuria,Childhood onset,Mild hearing impairment |
| NM_001134665.2(TRMT10A):c.697_698delinsTA | p.(Pro233*) | Hom | LP | Microcephaly, short stature, and impaired glucose metabolism type 1 | AR | Microcephaly,Blepharospasm,Delayed speech and language development,Seizures,Global developmental delay,Generalized myoclonic seizures,Febrile seizures,Disturbance of facial expression,Cyanotic episode |
| NM_001197104.1(KMT2A):c.2354_2402dup | p.(His801Glnfs*6) | Het | LP | Wiedemann-Steiner syndrome | AD | Enlarged kidney,Renal cyst,Polycystic kidney dysplasia,Low-set ears,Anteverted nares,Short neck,Premature birth,Abnormality of the cardiovascular system,Patent ductus arteriosus,Abnormal facial shape,Congenital onset,Depressed nasal bridge |
| NM_001159772.1(CANT1):c.902_906dup | p.(Ser303Alafs*21) | Hom | P | Desbuquois dysplasia type 1 | AR | Macrocephaly,Anteverted nares,Narrow chest,Edema,Abnormal facial shape,Skeletal dysplasia,Short nose,Coronal cleft vertebrae,Midface retrusion |
| NM_007317.2(KIF22):c.443C>T | p.(Pro148Leu) | Het | P | Spondyloepimetaphyseal dysplasia | AD | Abnormal facial shape,Respiratory distress,Skeletal dysplasia,Epiphyseal dysplasia,Hip dislocation,Short long bone,Short stature,Joint hyperflexibility,Broad phalanges of the 3rd finger,Short distal phalanx of finger,Feeding difficulties,Flat face |
| Chr5(SMN1):g.70241893-70247821del | p.? | Hom | P | Spinal muscular atrophy type 1 | AR | Motor delay,Muscle weakness,Gowers sign,EMG: neuropathic changes,Difficulty climbing stairs,Spinal muscular atrophy,Lower limb muscle weakness,Mildly elevated creatine kinase,Difficulty running,Childhood onset,Decreased patellar reflex,Toe walking |
| NM_006302.2(MOGS):c.1422G>A | p.(Trp474*) | Hom | LP | Congenital disorder of glycosylation type 2b | AR | Cryptorchidism,Cleft palate,Microcephaly,Micrognathia,Ptosis,Blepharophimosis,Arachnodactyly,Seizures,Muscular hypotonia,Tremor,Talipes equinovarus,Poor suck,Choroid plexus cyst,Head tremor,Cachexia,Fixed facial expression,Pes valgus,Camptodactyly |
| NM_000487.5(ARSA):c.583del | p.(Trp195Glyfs*5) | Hom | P | Metachromatic leukodystrophy | AR | Agenesis of corpus callosum,Developmental regression,Leukodystrophy,Difficulty standing,CNS demyelination,Abnormal myelination |
| NM_003742.2(ABCB11):c.2095T>C | p.(Ser699Pro) | Hom | LP | Familial intrahepatic cholestasis type 2 | AR | Delayed speech and language development,Jaundice,Pruritus,Global developmental delay,Abnormality of the liver,Abnormality of metabolism/Homeostasis,Hyperbilirubinemia,Elevated hepatic transaminase,Acholic stools |
| NM_020451.2(SELENON):c.872+1G>A | p.? | Hom | LP | *SEPN*-related myopathy | AR | Seizures,Muscular hypotonia,Global developmental delay |
| NM_000104.3(CYP1B1):c.1405C>T | p.(Arg469Trp) | Hom | P | Primary open angle congenital glaucoma type 3A | AR | Glaucoma,Cataract,Nystagmus,Autismt,Global developmental delay,Failure to thrive,Attention deficit hyperactivity disorder |
| NM_025137.3(SPG11):c.6437_6438del | p.(Thr2146Argfs*2 | Hom | LP | *SPG11*-related disorder | AR | Specific learning disability,Hypoplasia of the corpus callosum,Action tremor,Lower limb hyperreflexia,Leukodystrophy,Abnormality of the periventricular white matter,Babinski sign,Attention deficit hyperactivity disorder,Lower limb muscle weakness |
| NM_006984.4(CLDN10):c.653del | p.(Pro218Leufs*21) | Hom | LP | HELIX syndrome | AR | Hydronephrosis,Abnormality of the dentition,Hypertelorism,Strabismus,Visual impairment,Thick eyebrow,Carious teeth,Delayed speech and language development,Dry skin,Hirsutism,Global developmental delay,Specific learning disability,Failure to thrive,Hoarse voice,Abnormal facial shape,Hypophosphatemia,Delayed gross motor development,Hypomagnesemia,Skeletal muscle atrophy,Decreased body weight,Temperature instability,Abnormal autonomic nervous system physiology,Palmoplantar scaling skin |
| NM_000035.3(ALDOB):c.1013C>T | p.(Ala338Val) | Het | P | Hereditary fructose intolerance | AR | Renal insufficiency,Narrow mouth,Small face,Micrognathia,Low-set ears,Hypertonia,Limited elbow extension,Hepatic failure,High pitched voice,Poor suck,Elevated hepatic transaminase,Elbow flexion contracture,Abnormality of the patella,Ulnar deviation of the wrist,Short nose,Limited knee flexion/extension,Corneal opacity,Myotonia of the face,Flexion contracture of digit,Hand dimples |
| NM_000035.3(ALDOB):c.524C>A | p.(Ala175Asp) | Het | P | Hereditary fructose intolerance | AR | Same patient as above |
| NM_016035.3(COQ4):c.437T>G | p.(Phe146Cys) | Hom | P | Primary Coenzyme Q10 deficiency type 7 | AR | Intellectual disability,Seizures,Motor delay,Cerebellar atrophy,Cortical dysplasia,Scoliosis,Intellectual disability, severe,Widened subarachnoid space,Abnormal brain lactate level by MRS |
| NM_002495.2(NDUFS4):c.316C>T | p.(Arg106*) | Hom | P | *NDUFS4*-related disorder | AR | Recurrent urinary tract infections,Hypospadias,Poor eye contact,Seizures,Hypertrophic cardiomyopathy,Hyperglycemia,Myopathy |
| NM_001396.3(DYRK1A):c.572_575del | p.(Lys191Thrfs*6) | Het | LP | Mental retardation type 7 | AD | Microcephaly,Cafe-au-lait spot,Intellectual disability,Seizures,Global developmental delay,Atrial septal defect,Patent foramen ovale,Polymicrogyria,Febrile seizures,Aplasia/Hypoplasia of the cerebellum,Few cafe-au-lait spots |
| Chr12(GNPTAB):g.102158380-102158381del | p.? | Het (CH) | LP | Mucolipidosis type II alpha/beta | AR | Microcephaly,Abnormality of the skeletal system,Failure to thrive,Intrauterine growth retardation,Atrial septal defect,Abnormal facial shape,Abnormal bone structure,Abnormality of lysosomal metabolism |
| NM_024312.4(GNPTAB):c.2915+4_2915+9del | p.? | Het (CH) | LP | Mucolipidosis type II alpha/beta | AR | Same patient as above |
| NM_007171.3(POMT1):c.2167dup | p.(Asp723Glyfs*8) | Het | P | Muscular dystrophy-dystroglycanopathy (congenital with brain and eye anomalies), type A1 | AR | Parent of index with suspected ciliopahty |
| NM_025137.3(SPG11):c.1877del | p.(Phe626Serfs*7) | Het (CH) | LP | Spastic paraplegia type 11 | AR | Intellectual disability,Muscular hypotonia,Motor delay,Gait disturbance,Elevated serum creatine kinase,EMG: myopathic abnormalities,Proximal muscle weakness,Brain atrophy |
| NM_025137.3(SPG11):c.4852G>T | p.(Glu1618*) | Het (CH) | LP | Spastic paraplegia type 11 | AR | Same patient as above |
| NM_183075.2(CYP2U1):c.311_336dup | p.(Val113Argfs*7) | Hom | LP | Spastic paraplegia type 56 | AR | Developmental regression,Abnormality of the cerebral white matter |
| NM_017799.3(TMEM260):c.1698_1701del | p.(Tyr567Thrfs*27) | Hom | P | Structural heart defects and renal anomalies syndrome | AR | Delayed speech and language development,Intellectual disability,Global developmental delay,Motor delay |
| NM_001042492.2(NF1):c.2998_2999del | p.(Arg1000Cysfs*20) | Het | LP | Neurofibromatosis type 1 | AD | Axillary freckling,Abnormality of skin pigmentation,Neonatal onset,Multiple cafe-au-lait spots |
| NM_001346813.1(ARID1B):c.1380_1398del | p.(Gly461Serfs*35) | Het | LP | Coffin-Siris syndrome type 1 | AD | Poor eye contact,Hypertrichosis,Slender finger,Seizures,Global developmental delay,Cerebellar hypoplasia,Long hallux,Hypoplasia of the corpus callosum,Low posterior hairline,Curly hair,Cavum septum pellucidum,Cerebral venous angioma,Dysgenesis of the hippocampus |
| NM_001267550.1(TTN):c.2224del | p.(Ser742Profs*5) | Het | LP | TTN-related disorder | AD | Muscle weakness,Fatigable weakness of skeletal muscles,Abnormality of skeletal muscles |
| Chr5(SMN1):g.70241893_70247821del | p.? | Hom | P | Spinal muscular atrophy type 1 | AR | Small face,Long face,Abnormality of the midface,Muscular hypotonia,Spasticity,Areflexia,Tongue fasciculations,Muscle weakness,Flexion contracture,Abnormal facial shape,Difficulty walking,Scoliosis,Infantile onset,Muscular hypotonia of the trunk,Contracture of the distal interphalangeal joint of the fingers,Flexion contracture of finger,Lingual dystonia |
| NM_003829.4(MPDZ):c.3253A>T | p.(Lys1085*) | Hom | LP | Nonsyndromic hydrocephalus type 2 | AR | Ventricular septal defect,Atrial septal defect,Secundum atrial septal defect,Ventriculomegaly,Abnormality of the septum pellucidum,Antenatal onset |
| NM_019098.4(CNGB3):c.1148del | p.(Thr383Ilefs*13) | Hom | P | CNGB3-related achromatopsia type 3 | AR | Cone-rod dystrophy |
| NM_001323544.1(GALNS):c.1037G>A | p.(Gly346Asp) | Het (with het VUS) | P | Mucopolysaccharidosis type IVA | AR | Scoliosis,Abnormality of the acetabulum,Difficulty climbing stairs,Absent patellar reflexes,Difficulty running |
| NM_000518.4(HBB):c.316-106C>G | p.? | Het | P | Beta-thalassemia (minor) | AD | Delayed speech and language development,Seizures,Muscular hypotonia,Motor delay,Short foot,Hypoplasia of the corpus callosum,Ventriculomegaly,EEG abnormality,Intellectual disability, severe,Small hand |
| NM_000262.2(NAGA):c.973G>A | p.(Glu325Lys) | Het (with het VUS) | P | NAGA deficiency | AR | Delayed speech and language development,Seizures,Muscular hypotonia,Motor delay,Short foot,Hypoplasia of the corpus callosum,Ventriculomegaly,EEG abnormality,Intellectual disability, severe,Small hand |
| NM_014795.3(ZEB2):c.1683_1689dup | p.(Asp564Phefs*7) | Het | LP | Mowat-Wilson syndrome | AD | Abnormality of the midface,Hypertelorism,Strabismus,Global developmental delay,Abnormal corpus callosum morphology,Congenital onset,Sporadic,Muscular hypotonia of the trunk |
| NM_005629.3(SLC6A8):c.1255-35_1272del | p.? | Het | LP | Cerebral creatine deficiency syndrome type 1 | XL | Muscular hypotonia,Encephalopathy,Small for gestational age,Abnormal facial shape,Neonatal onset |
| NM_001242896.1(DEPDC5):c.3463_3465del | p.(Ser1155del) | Het | LP | Familial focal epilepsy with variable foci | AD | Same patient as above |
| NM_000402.3(G6PD):c.653C>T | p.(Ser218Phe) | Hem | P | Hemolytic anemia due to G6PD deficiency | XL | Optic nerve hypoplasia,Seizures,Hyperbilirubinemia,Focal-onset seizure,Abnormality of retinal pigmentation |
| NM_177400.2(NKX6-2):c.196del | p.(Arg66Glyfs*122) | Hom | P | Spastic ataxia type 8, with hypomyelinating leukodystrophy | AR | Hearing impairment,Visual impairment,Intellectual disability,Seizures,Muscular hypotonia,Global developmental delay,Leukodystrophy,Jerky head movements,Corpus callosum atrophy,Central hypotonia |
| NM_005859.4(PURA):c.812_814del | p.(Phe271del) | Het | P | Mental retardation type 31 | AD | Abnormality of the philtrum,Intellectual disability,Muscular hypotonia,Global developmental delay,Gait disturbance,Sandal gap,CNS hypomyelination,Cognitive impairment |
| ChrX(PLP1):g.96607678_106835457dup | p.? | Hem | P | Pelizaeus-Merzbacher disease | XL | Strabismus,Nystagmus,Muscular hypotonia,Motor delay,Hyperreflexia,Intrauterine growth retardation,Leukodystrophy,Congenital onset,Pendular nystagmus,Brain atrophy,Abnormal myelination |
| Chr17(COX10): g.14097117-15422835del | p.? | Het (with het VUS) | P | Mitochondrial COX4 deficiency | AR | Hearing impairment,Sensorineural hearing impairment,Abnormal erythrocyte morphology,Eosinophilia,Anemia,Renal tubular acidosis,Increased serum lactate,Gastrointestinal hemorrhage,Colitis,Elevated serum creatine kinase,Increased serum pyruvate,Bilineage myelodysplasia,Abnormal myeloid cell morphology |
| NM_000214.2(JAG1):c.2278del | p.(Val760Trpfs*60) | Het | LP | JAG1-related disorder | AD | Inguinal hernia,Hypospadias,Broad forehead,Abnormality of the nasal tip,Congenital hypothyroidism,Intellectual disability,Intellectual disability, mild,Global developmental delay,Limited elbow extension,Failure to thrive,Fetal ascites,Abnormal facial shape,Gastroesophageal reflux,Curly hair,Poor appetite,Pulmonary artery stenosis,Sparse hair,Thick nasal alae,Congenital posterior urethral valve,Thick vermilion border,Abnormality of bladder morphology,Impaired toileting ability,Hernia |
| NM_001849.3(COL6A2):c.1615C>T | p.(Arg539*) | Het | LP | Ullrich congenital muscular dystrophy 1 | AD | Same patient as above |
| NM_001110792.1(MECP2):c.1440dup | p.(Pro481Alafs*18) | Hem | LP | X-linked syndromic Metal retardation Lubs type | XL | Micrognathia,Strabismus,Stereotypy,Global developmental delay,Absent speech,Joint hypermobility,Gowers sign |
| NM_000543.4(SMPD1):c.1624C>T | p.(Arg542*) | Hom | P | Niemann-Pick disease type A/B | AR | Abnormality of the nervous system,Intellectual disability,Splenomegaly,Hepatomegaly |
| NM_000271.4(NPC1):c.2777C>T | p.(Ala926Val) | Hom | P | Niemann-Pick disease type C1 | AR | Supranuclear gaze palsy,Abnormality of the nervous system,Intellectual disability,Failure to thrive,Splenomegaly,Vomiting,Hepatomegaly,Developmental regression |
| NM_000271.4(NPC1):c.1628del | p.(Pro543Argfs*20) | Hom | P | Niemann-Pick disease type C1 | AR | Abnormality of the nervous system,Intellectual disability,Failure to thrive,Failure to thrive in infancy,Splenomegaly,Vomiting,Hepatomegaly,Elevated hepatic transaminase,Abdominal distention,Neonatal onset,Prolonged neonatal jaundice,Mild global developmental delay |
| NM_000543.4(SMPD1):c.847G>A | p.(Ala283Thr) | Hom | P | Niemann-Pick disease A/B | AR | Splenomegaly,Hepatomegaly |
| NM_012327.5(PIGN):c.963G>A | p.(Gln321=) | Het (with het VUS) | P | Multiple congenital anomalies-hypotonia-seizures syndrome-1 | AR | Aniridia,Abnormality of the respiratory system,Respiratory failure,Congenital onset |
| NM_001101.4(ACTB):c.625G>A | p.(Val209Met) | Het | LP | Baraitser-Winter syndrome type 1 | AD | Wide mouth,High palate,Thin upper lip vermilion,Epicanthus,Smooth philtrum,Narrow forehead,Long philtrum,Broad nasal tip,Webbed neck,Upslanted palpebral fissure,Pectus carinatum,Hypertrichosis,Intellectual disability,Abnormal umbilicus morphology,Premature birth,Low posterior hairline,Highly arched eyebrow,High, narrow palate,Cubitus valgus,Hyperlordosis,Congenital onset,Short stature,Prominent metopic ridge,Attention deficit hyperactivity disorder,Partial duplication of thumb phalanx,Increased nuchal translucency,Abnormal temper tantrums,Palpebral edema |
| NM_014297.3(ETHE1):c.554T>G | p.(Leu185Arg) | Hom | P | Ethylmalonic encephalopathy | AR | Fetus (ongoing pregnancy). Family history of sibling deceased at the age of 11 months, suspected with a metabolic disease, mitochondrial disease or pyruvate dehydrogenase deficiency. |
| NM_005045.3(RELN):c.9444-1G>A | p.? | Het | LP | Epilepsy, familial temporal lobe, 7 | AD | Behavioral abnormality,Autism,Obsessive-compulsive behavior,Stereotypy,Impaired social interactions,Anxiety,Delayed speech and language development,Poor eye contact,Eczema,Intellectual disability,Encephalopathy,EEG abnormality,Developmental regression,Attention deficit hyperactivity disorder,Motor tics,Insomnia |
| NM_001321544.1:c.871A>G | p.(Asn291Asp) | Hom | LP | SLC25A42-related disorder | AR | Visual impairment,Blindness,Nystagmus,Ataxia,Lethargy,Spasticity,Motor delay,Hyperreflexia,Intrauterine growth retardation,Abnormality of the cerebrum,Abnormality of the basal ganglia,Delayed gross motor development,Leukoencephalopathy,Lower limb hyperreflexia,Leukodystrophy,Poor head control,Generalized muscle weakness,Metachromatic leukodystrophy variant,Upper limb spasticity,Deep white matter hypodensities,Peripheral neuropathy,Abnormal CNS myelination,Abnormality of the anterior pituitary,Abnormal myelination,Delayed myelination,Abnormality of the brainstem white matter,Ectopic anterior pituitary gland,Abnormal basal ganglia MRI signal intensity,Cerebral white matter atrophy,Abnormal brain lactate level by MRS,Increased size of nasopharyngeal adenoids |
| NM_003560.2(PLA2G6):c.2370T>G | p.(Tyr790*) | Hom | P | PLA2G6-related disorder | AR | Cerebellar atrophy,Abnormality of the cerebral white matter,Abnormality of the periventricular white matter,Cerebral hypomyelination,Corpus callosum atrophy,Punctate periventricular T2 hyperintense foci,Periventricular white matter hyperdensities |
| NM_004006.2(DMD):c.1283del | p.(Asn428Ilefs*9) | Hem | LP | Duchenne muscular dystrophy | XL | Muscular hypotonia,Myopathy,Muscular dystrophy,Abnormal circulating creatine kinase concentration |
| NM_001126131.1(POLG):c.911T>G | p.(Leu304Arg) | Hom | P | POLG-related disorders | AR | Ptosis,Ophthalmoplegia,Abnormality of the scapula,Muscular hypotonia,Muscle weakness,Scoliosis,Myopathy,Skeletal muscle atrophy |
| NM_001244810.1(FOXP1):c.1103dup | p.(His368Glnfs*93) | Het | LP | Mental retardation with language impairment and with or without autistic features | AD | Behavioral abnormality,Autism,global developmental delay,Poor motor coordination |
| NM_020320.3(RARS2):c.1A>T | p.? | Het (with het LP) | P | Pontocerebellar hypoplasia type 6 | AR | Delayed speech and language development,Abnormality of Krebs cycle metabolism,Muscular hypotonia,Global developmental delay,Motor delay,Failure to thrive,Anemia,Vomiting,Increased CSF lactate,Hypoventilation,Lactic acidosis,Hyperalaninemia,Abnormal circulating pyruvate family amino acid concentration |
| chr6(RARS2):g.88253327-88253723dup | p.? | Het (with het P) | LP | Pontocerebellar hypoplasia type 6 | AR | Same patient as above |
| NM_022370.3(ROBO3):c.3412del | p.(Arg1138Glufs*101) | Het | LP | Gaze palsy, familial horizontal, with progressive scoliosis, 1 | AR | Abnormality of the head,Generalized hypotonia,Abnormal facial shape,Craniofacial asymmetry |
| NM_022370.3(ROBO3):c.767-1G>A | p.? | Het | LP | Gaze palsy, familial horizontal, with progressive scoliosis, 1 | AR | Same patient as above |
| NM_000051.3(ATM):c.6154G>A | p.(Glu2052Lys) | Het | P | Breast cancer, susceptibility to | AD | Breast carcinoma,Ductal carcinoma in situ |
| NM_000152.3(GAA):c.-32-13T>G | p.? | Hom | P | Glycogen storage disease type II | AR | Long philtrum,Lacrimal duct atresia,Aplasia cutis congenita,Frontal bossing,Intracranial hemorrhage,Difficulty walking,Congenital onset,Aplasia cutis congenita of scalp,Prominent ear helix,Localized skin lesion,Cephalohematoma |
| NM_016381.3(TREX1):c.309dup | p.(Thr104Hisfs*53) | Hom | P | Aicardi-Goutieres syndrome type 1 | AR | Microcephaly,Visual impairment,Intellectual disability,Muscular hypotonia,Lethargy,Spasticity,Motor delay,Hypertonia,Encephalopathy,Hyperreflexia,EEG abnormality,Leukodystrophy,Abnormality of the cerebral cortex,Infantile muscular hypotonia,Intracranial cystic lesion,Abnormal myelination |
| NM_001256442.1(PRRT2):c.649dup | p.(Arg217Profs*8) | Het | P | PRRT2-related disorder | AD | Intellectual disability, mild,Global developmental delay,Generalized-onset seizure |
| NM_001258462.1(PAX6):c.11-218_565+80del | p.? | Het | P | Aniridia | AD | Coloboma,Nystagmus,Delayed speech and language development,Global developmental delay,Motor delay,Agenesis of corpus callosum,Growth delay,Delayed gross motor development,Brain atrophy,Abnormality of the optic disc |
| NM_014141.5(CNTNAP2):c.498G>A | p.(Trp166*) | Het (with het LP) | LP | Pitt-Hopkins like syndrome 1 | AR | Micrognathia,Hypotelorism,Intellectual disability,Seizures |
| NM_014141.5(CNTNAP2):c.5_8dup | p.(Ala4Glyfs*34) | Het (with het LP) | LP | Pitt-Hopkins like syndrome 1 | AR | Same patient as above |
| NM_139058.2(ARX):c.315_335dup | p.(Ala109_Ala115dup) | Hem | P | ARX-associated developmental disorder | XL | Seizures,Global developmental delay,Generalized hypotonia,Dystonia,Poor head control,Generalized limb muscle atrophy,Central hypotonia,Dyskinesia |
| NM_001243226.2(TCF4):c.2045G>A | p.(Arg682Gln) | Het | P | Pitt-Hopkins syndrome | AD | Wide mouth,Abnormal lip morphology,Seizures,Global developmental delay,Absent speech,Inability to walk,Intermittent hyperventilation,Apneic episodes in infancy,Cyanotic episode |
| NM_015910.6(WDPCP):c.253+2T>C | p.? | Het (with het VUS) | LP | Bardet -Biedl syndrome type 15 | AR | Small anterior fontanelle,Preauricular skin tag,Irritability,Growth delay,Intrauterine growth retardation,Premature birth,Patent ductus arteriosus,Patent foramen ovale,Abnormal facial shape,Poor suck,Apnea,Poor head control,Hyperbilirubinemia,Tricuspid regurgitation,Abnormality of the renal pelvis,Left-to-right shunt |
| NM_000836.2(GRIN2D):c.1724C>T | p.(Ser575Leu) | Het | LP | Early infantile epileptic encephalopathy type 46 | AD | Behavioral abnormality,Abnormality of skin pigmentation,Hypopigmentation of the skin,Seizures,Absence seizure,EEG abnormality,Expressive language delay,Neonatal onset,Episodic respiratory distress,Loss of consciousness,Atypical absence seizure,Hyperpigmented/hypopigmented macules,Mild receptive language delay |
| NM_023035.2(CACNA1A):c.674C>G | p.(Pro225Arg) | Het | LP | Early infantile epileptic encephalopathy type 42 | AD | Cryptorchidism,Difficulty in tongue movements,Behavioral abnormality,Self-mutilation,Delayed speech and language development,Poor eye contact,Seizures,Choreoathetosis,Motor delay,Poor suck,Broad-based gait,Gait imbalance,Abnormality of the cerebellar vermis,Difficulty walking,Poor head control,Infantile onset,Neonatal onset,Involuntary movements,Intellectual disability, severe,Cerebellar hemisphere hypoplasia |
| NM_001141945.2(ACTA2):c.536G>A | p.(Arg179His) | Het | P | ACTA2-related disorder | AD | Delayed speech and language development,Intellectual disability,Seizures,Global developmental delay,Motor delay,Stroke,Ventricular septal defect,Patent foramen ovale,Ischemic stroke,Abnormality of the cerebral white matter,Abnormality of the periventricular white matter,Aortic aneurysm,Abnormal cerebral artery morphology,Abnormal cutaneous elastic fiber morphology |
| Chr2(NPHP1):g.110827496-110962791del | p.? | Hom | LP | Joubert syndrome type 4 | AR | Polyuria,Renal cyst,Renal tubular dysfunction,Nystagmus,Delayed speech and language development,Hypertension,Ataxia,Global developmental delay,Motor delay,Failure to thrive,Anemia,Polydipsia,Vomiting,Short stature |
| NM_020366.3(RPGRIP1):c.1107del | p.(Glu370Asnfs*5) | Hom | P | Leber congenital amaurosis type 6 | AR | Hyperopic astigmatism,Strabismus,Visual impairment,Visual loss,Nystagmus,Optic atrophy,Infantile onset |
| NM_001330437.1(PTPN11):c.922A>G | p.(Asn308Asp) | Het | P | Noonan syndrome type 1 | AD | Optic nerve hypoplasia,Optic atrophy,Cerebellar vermis hypoplasia,Cerebellar hypoplasia |
| NM_000117.2(EMD):c.82+1G>A | p.? | Hem | LP | Emery-Dreifuss muscular dystrophy 1 | XL | Progressive muscle weakness |
| NM_019066.4(MAGEL2):c.1808C>G | p.(Ser603*) | Het | LP | Schaaf-Yang syndrome | AD | Vesicoureteral reflux,Global developmental delay,Motor delay,Abnormal facial shape,Frontal bossing,Delayed gross motor development,Inability to walk,Short stature,Short finger,Childhood onset |
| NM_001042492.2(NF1):c.5305C>T | p.(Arg1769*) | Het | P | Neurofibromatosis type 1 | AD | Cafe-au-lait spot,Neurofibromas,Congenital onset,Sporadic,Fibroma |
| NM_000256.3(MYBPC3):c.1678del | p.(Asp560Thrfs*19) | Het | P | Familial hypertrophic cardiomyopathy type 4 | AD | Abnormal myocardium morphology,Hypertrophic cardiomyopathy,Abnormal cardiac septum morphology,Ventricular tachycardia,Antenatal onset |
| NM_005807.4(PRG4):c.2247del | p.(Ala750Leufs*162) | Hom | LP | Camptodactyly -arthropathy-coxa varapericarditis syndrome | AR | Arthropathy,Polyarticular arthropathy,Childhood onset |
| NM_001286704.1(UFM1):c.-273_-271del | p.? | Hom | P | hypomyelinating leukodystrophy type 14, | AR | Microcephaly,Hearing impairment,Seizures,Global developmental delay |
| NM_001849.3(COL6A2):c.1817-3C>G | p.? | Hom | P | COL6A2-associated myopathy | AR | Myopathy,EMG: myopathic abnormalities,Muscular dystrophy,Proximal muscle weakness,Childhood onset |
| NM_032119.3(ADGRV1):c.17518del | p.(Tyr5840Thrfs*102 | Hom | LP | Usher syndrome type 2C | AR | Bilateral sensorineural hearing impairment; Failure to thrive; Hearing impairment; Muscular hypotonia; Progressive external ophthalmoplegia; Proximal muscle weakness |
| NM_003901.3(SGPL1):c.665G>A | p.(Arg222Gln) | Hom | P | Nephrotic syndrome type 14 | AR | Same patient as above |

**Table 3. Intronic VUS reported in patients with GS**

| Variant | Variant coordinates | Zygosity | Patient HPOs | OMIM phenotype | Supporting evidence |
| --- | --- | --- | --- | --- | --- |
| NM_014946.3(*SPAST*):c.587-608_587-557del | NC_000002.11:g.32323257_32323308del | Het | Hearing impairment,Intellectual disability,Ataxia,Spasticity,Hypertonia,Infantile onset,Short stature,Progressive gait ataxia | Spastic paraplegia type 4, AD (OMIM®:182601) | Novel, co-segregating in 2 affected siblings, parents not tested. |
| NM_018706.6(*DHTKD1*):c.2402+3A>G | NC_000010.10:g.12159757A>G | CH with exonic VUS | Intellectual disability,Hepatosplenomegaly,Anemia | 2-aminoadipic 2-oxoadipic aciduria, AR (OMIM®:614984) | Very rare, predicted to affect splicing, co-segregating in affected sibling |
| NM_001287.5(*CLCN7*):c.739-18G>A | NC_000016.9:g.1507356C>T | Hom | Abnormality of vision,Recurrent fractures,Fractures of the long bones,Osteopetrosis | Osteopetrosis type 4, AR (OMIM®:602727) | PMID: 29620724, detected in an unrelated patient in CentoMD® with radiological findings suggestive of osteopetrosis |
| NM_001128431.3(*SLC39A14*):c.751-9C>G | NC_000008.10:g.22273273C>G | Hom | Behavioral abnormality,Spasticity,Spastic paraplegia,Global developmental delay,Overgrowth,Dysphagia,Constipation,Neurodegeneration,Frequent falls,Inability to walk,Muscle stiffness,Poor appetite,Lumbar kyphosis,Limb pain,Feeding difficulties,Abnormality of brain morphology,Abnormality of movement | Hypermanganesemia with dystonia type 2, AR (OMIM®:617013) | Predicted to affect splicing, novel |
| NM_001005373.3(*LRSAM1):*c.1600-106A>G | NC_000009.11:g.130257493A>G | Hom | Muscle weakness | Charcot-Marie-Tooth disease axonal type 2P, AR (OMIM®:614436) | Predicted to create a new splicing site, novel |
| NM_014795.3(*ZEB2*):c.74-15372C>A | NC_000002.11:g.145202965G>T | Het | Microcephaly,Hypodontia,Conical tooth,Delayed speech and language development,Abnormal external genitalia,Lymphedema,Intellectual disability,Seizures,Muscular hypotonia,Abnormal facial shape,Constipation,Arnold-Chiari malformation,Developmental regression,Decreased body weight,Focal-onset seizure,Abnormal myelination,Cerebral visual impairment | Mowat-Wilson syndrome, AD (OMIM®:235730) | Variant located in a highly conserved regulatory region in intron 2, novel |
| NM_000531.5(*OTC)*:c.-366A>G | NC_000023.10:g.38211584A>G | Hem | Autism,Delayed speech and language development,Cafe-au-lait spot,Intellectual disability,Elevated hepatic transaminase,Elevated serum creatine kinase,Abnormal circulating aspartate family amino acid concentration | Ornithine transcarbamylase deficiency, XL (OMIM®:311250) | PMID: 20127982 |
| NM_152773.4(*TCTEX1D2*):c.113+6T>C | NC_000003.11:g.196044905A>G | Hom | Abnormal cerebellum morphology,Intrauterine growth retardation,Pericardial effusion,Talipes equinovalgus,Enlarged cisterna magna,Skeletal dysplasia,Short long bone,Short femur,Thoracic hypoplasia,Rhizomelia,Limb undergrowth,Polydactyly | Short-rib thoracic dysplasia type 17, AR (OMIM®:617353) | Predicted to disrupt the donor splice site of intron 1, novel |
| NM_002618.3(*PEX13*):c.*1564_*1566delinsGCT | NC_000002.11:g.61277469_61277471delinsGCT | Hom | Cataract,Intellectual disability,Hypertonia,Hyperreflexia,Progressive spasticity,Developmental regression,Leukodystrophy,Attention deficit hyperactivity disorder,Brain atrophy,Abnormal myelination | Peroxisome biogenesis disorder type 11B, AR (OMIM®:614885) | Delins in 3' region, novel |
| NM_018684.3(*ZC4H2*):c.225+5G>A | NC_000023.10:g.64141692C>T | Hem | Muscular hypotonia,Failure to thrive,Developmental regression | Wieacker-Wolff syndrome, XL (OMIM®:314580) | PMID: 26056227, disrupt the highly conserved donor splice site of exon 2 |
| NM_000138.4(*FBN1*):c.539-9450A>G | NC_000015.9:g.48839455T>C | Het | Failure to thrive,Aortic root aneurysm,Macular hyperpigmentation,Dilatation of the sinus of Valsalva | Marfan syndrome, AD (OMIM®:154700) | Predicted to affect splicing, novel |
| NM_001348748.1(*DENND5A*):c.3387+3G>T | NC_000011.9:g.9164947C>A | Hom | Microcephaly,Visual impairment,Delayed speech and language development,Seizures,Muscular hypotonia,Global developmental delay,Hypoplasia of the corpus callosum,Abnormality of the cerebral white matter | Early infantile epileptic encephalopathy type 49, AR (OMIM®:617281) | Predicted to affect splicing, novel |
| NM_001205254.1(*OCLN*):c.1037+1G>A | NC_000005.9:g.68830667G>A | Hom | Brachycephaly,Microcephaly,Visual impairment,Esotropia,Optic atrophy, Hypothyroidism,Abnormality of skin pigmentation,Intellectual disability,Seizures,Muscular hypotonia,Spasticity,Global developmental delay,Agenesis of corpus callosum,Dystonia,Failure to thrive,Intrauterine growth retardation,Basal ganglia calcification,Generalized-onset seizure,Brain atrophy | Pseudo-TORCH syndrome type 1, AR (OMIM®:251290) | Affecting canonical splicing site but one Hom patient in CentoMD® with different phenotype, one Hom adult individual in gnomAD |
| NM_000393.4(*COL5A2*):c.4114-10_4114-7del | NC_000002.11:g.189899888_189899891del | Het | Strabismus,Exotropia,Bruising susceptibility,Arachnodactyly,Cholestasis,Disproportionate tall stature,Slender build,Mitral valve prolapse,Weight loss,Vomiting,Diarrhea,Nausea,Gastroesophageal reflux,Scoliosis,Chronic pancreatitis,Keloids,Allergy,Neoplasm of the colon | Ehlers-Danlos syndrome, classic type 2, AD (OMIM®:130010) | Predicted to affect the highly conserved acceptor splice site of exon 53, novel |
| NM_078470.5(*COX15*):c.750+85A>G | NC_000010.10:g.101483628T>C | Hom | Amblyopia,Global developmental delay,Abnormality of the basal ganglia | Leigh syndrome due to cytochrome c oxidase deficiency, AR (OMIM®:256000) | Co-segregating in affected sibling, very rare |
| NM_004924.3(*ACTN4*):c.733+4A>G | NC_000019.9:g.39200120A>G | Het | Abnormality of the kidney,Renal insufficiency,Abnormality of the renal tubule | Focal segmental glomerulosclerosis type 1, AD (OMIM®:604638) | Predicted to affect splicing, novel |
| NM_005573.3(*LMNB1*):c.359+6T>C | NC_000005.9:g.126113565T>C | Hom | Abnormality of the kidney,Brachycephaly,Microcephaly,Smooth philtrum,Prominent nasal bridge,Cutis marmorata,Muscular hypotonia,Global developmental delay,Abnormal facial shape,Frequent falls,Prominent ear helix,Prominent forehead,Midface retrusion | LMNB1-associated disorder, AD (OMIM®:169500) | Predicted to affect splicing, novel. Hom for AD disorder |
| NM_001142784.2(*IL11RA*):c.331+6T>G | NC_000009.11:g.34656911T>G | Hom | Abnormal facial shape,Recurrent upper respiratory tract infections,Multiple suture craniosynostosis,Pansynostosis | Craniosynostosis and dental anomalies, AR (OMIM®:614188) | Predicted to affect splicing, novel |
| NM_003384.2(*VRK1*):c.-5-146A>G ^1^ | NC_000014.8:g.97299658A>G | Hom | Hydronephrosis,Microcephaly,Failure to thrive,Premature birth,Patent foramen ovale,Premature rupture of membranes,Abnormal facial shape,Dyspnea,Apnea,Hypokalemia,Hyponatremia,Decreased body weight,Elevated diastolic blood pressure,Pulmonary insufficiency,Feeding difficulties | Pontocerebellar hypoplasia type 1A, AR (OMIM®: 607596) | Predicted to affect splicing, novel |
| NM_004211.3(*SLC6A5*):c.3+5G>A | NC_000011.9:g.20621226G>A | CH with exonic VUS | Nystagmus,Intellectual disability,Muscular hypotonia,Spasticity,Global developmental delay,Spastic diplegia,Motor delay,Hyperreflexia,Joint hypermobility,Growth delay,Fever,Abnormal facial shape,Poor head control,Scissor gait | Hyperekplexia type 3, AR (OMIM®:614618) | Predicted to affect splicing, novel |
| NM_001080420.1(*SHANK3*):c.600+4A>G | NC_000022.10:g.51117352A>G | Het | Macrocephaly,Cataract,Delayed speech and language development,Cutis laxa,Global developmental delay,Motor delay,Joint laxity,Developmental regression | Phelan-McDermid syndrome, AD (OMIM®:606232) | Predicted to disrupt the highly conserved donor splice site of exon 5, novel |
| NM_001206999.1(*CIT*):c.3350+5G>A | NC_000012.11:g.120171964C>T | CH with exonic VUS | High palate,Microcephaly,Retrognathia,Microretrognathia,Triangular face,Posteriorly rotated ears,Congenital hypothyroidism,Failure to thrive,Intrauterine growth retardation,Oligohydramnios,Premature birth,Abnormal facial shape,High, narrow palate,Pathologic fracture,Skeletal muscle atrophy | Primary microcephaly type 17, AR (OMIM®:605629) | Predicted to disrupt the highly conserved donor splice site of exon 26 |
| NM_000182.4(*HADHA*):c.1480-139C>G | NC_000002.11:g.26418240G>C | Hom | Hypoparathyroidism,Seizures,Patent foramen ovale,Neutropenia,Iron deficiency anemia,Sideroblastic anemia,Metabolic acidosis,Generalized tonic-clonic seizures,Hypocalcemia,Hyperphosphatemia,Involuntary movements,Abnormal circulating fatty-acid concentration,Abnormality of alkaline phosphatase activity,Abnormality of the gallbladder,Abnormal circulating aspartate family amino acid concentration,Coarsened hepatic echotexture,Vitamin D deficiency,Decreased activity of 3-hydroxyacyl-CoA dehydrogenase | Trifunctional protein deficiency/Isolated deficiency of long-chain 3-hydroxyl-CoA dehydrogenase, AR (OMIM®:609015/609016) | Positive biochemical screening for trifunctional protein deficiency and LCHAD. Novel variant, not predicted to affect splicing |
| NM_000518.4(*HBB*):c.-31C>T | NC_000011.9:g.5248282G>A | Het in *cis* with a het P variant | Seizures,Muscular hypotonia,Short foot,Hypoplasia of the corpus callosum,Ventriculomegaly,EEG abnormality,Intellectual disability,Small hand | Beta-thalassemia (minor), AD (OMIM®:613985) | PMID: 19657842, 23425204, in cis with a P variant |
| NM_001185090.1(*GRIN1*):c.2396+5G>A | NC_000009.11:g.140057787G>A | Hom | Microcephaly,Muscular hypotonia,Global developmental delay,Failure to thrive,Abnormal visual fixation | Neurodevelopmental disorder with or without hyperkinetic movements and seizures, AR (OMIM®:138249) | Predicted to affect splicing, very rare |
| NM_015284.3(*SZT2*):c.2929+15C>T | NC_000001.10:g.43891635C>T | Hom | Hydronephrosis,Brachycephaly,Epicanthus,Micrognathia,Low-set ears,Single transverse palmar crease,Seizures,Muscular hypotonia,Global developmental delay,Obesity,Abnormal facial shape,Hypoplasia of the corpus callosum,Poor head control,Abnormal head movements,Highly arched eyebrow,Almond-shaped palpebral fissure,Muscular hypotonia of the trunk,Infantile spasms,Abnormality of movement,Moon facies | Early infantile epileptic encephalopathy type 18, AR (OMIM®: 615476) | Very rare, predicted to affect splicing, co-segregating in affected sibling |
| NM_001271043.2(*NFIX*):c.1278+698C>T | NC_000019.9:g.13193367C>T | Het | Macrocephaly,Autism,Cutis marmorata,Muscular hypotonia,Global developmental delay,Generalized hypotonia,Laryngomalacia,Premature birth,Anemia,Abnormal facial shape,Hepatomegaly,PsycHomotor deterioration,Recurrent pneumonia,Mesoaxial foot polydactyly | Sotos syndrome type 2, AD (OMIM®:614753) | Predicted to enforce cryptic splice site nearby, novel |
| NM_153252.4(*BRWD3*):c.3602+20C>G | NC_000023.10:g.79946532G>C | Hem | Hydronephrosis,Dolichocephaly,Facial asymmetry,Deeply set eye,Muscular hypotonia,Global developmental delay,Plagiocephaly,Polyhydramnios,Abnormal facial shape,Feeding difficulties in infancy,Fatigue,Delayed myelination,Stage 1 chronic kidney disease | Mental retardation type 93, XL (OMIM®:300553) | Predicted to affect splicing, novel |
| NM_001104631.1(*PDE4D*):c.456-171954_456-172120del | NC_000005.9:g.58683748_58683914del | Het | Behavioral abnormality,Aggressive behavior,Obsessive-compulsive behavior,Autistic behavior,Anxiety,Delayed speech and language development,Hyperactivity,Skin rash,Pruritus,Vomiting,Inflammation of the large intestine,Sleep disturbance,Autoimmunity,Abnormal social behavior,Inappropriate crying | Acrodysostosis with or without hormone resistance type 2, AD (OMIM®:614613) | ~170 bp deletion in intron 1 |
| NM_004006.2(*DMD*):c.6615-10949_6614+15437del | NC_000023.10:g.31961293_31971019del | Hem | Muscular hypotonia,Unsteady gait,Inability to walk,Scoliosis,Multiple joint contractures,Hyperlordosis,Muscular dystrophy | Duchenne muscular dystrophy, XL (OMIM®:310200) | ~9.7 kb del in intron 45, not listed in Decipher, only detected in female patients at CentoMD® |
| NM_001308153.1(*ARHGEF10*):c.1894-1G>T | NC_000008.10:g.1853736G> | Het | Abnormality of the coagulation cascade,Adult onset,Demyelinating peripheral neuropathy,Peripheral demyelination,Vitamin K deficiency | Slowed nerve conduction velocity, AD (OMIM®: 608236) | Affecting canonical splicing site, novel, but LoF is not clearly established as disease mechanism |
| NM_015910.6(*WDPCP*):c.1748+9431A>G | NC_000002.11:g.63596090T>C | Het-Het with LP variant | Small anterior fontanelle,Preauricular skin tag,Irritability,Intrauterine growth retardation,Premature birth,Patent ductus arteriosus,Patent foramen ovale,Abnormal facial shape,Poor suck,Apnea,Poor head control,Hyperbilirubinemia,Tricuspid regurgitation,Abnormality of the renal pelvis,Left-to-right shunt | Bardet -Biedl syndrome type 15, AR (OMIM®:615992) | Predicted to affect splicing, very rare |
| NM_001318507.1(*CTU2*):c.1086+5G>A | NC_000016.9:g.88779860G>A | Hom | Microcephaly,Seizures,Muscular hypotonia,Global developmental delay,Choreoathetosis,Small for gestational age,Developmental regression,Limb joint contracture | Microcephaly, facial dysmorphism, renal agenesis, and ambiguous genitalia syndrome, AR (OMIM®: 617057) | Predicted to affect splicing, novel |
| NM_014141.5(*CNTNAP2*):c.2099-79071A>G | NC_000007.13:g.147521586A>G | Het-Het with intronic VUS | Microcephaly,Seizures,Muscular hypotonia,Global developmental delay,Choreoathetosis,Small for gestational age,Developmental regression,Limb joint contracture | Cortical dysplasia-focal epilepsy syndrome, AR (OMIM®:610042) | Predicted to affect splicing, novel |
| NM_014141.5(*CNTNAP2*):c.98-105810_98-46301del | NC_000007.13:g.146365553_146425062del | Het-Het with intronic VUS | Microcephaly,Seizures,Muscular hypotonia,Global developmental delay,Choreoathetosis,Small for gestational age,Developmental regression,Limb joint contracture | Cortical dysplasia-focal epilepsy syndrome, AR (OMIM®:610042) | 59.5 kb deletion in intron 1, similar deletion reported in Decipher as VUS in a patient with NDD |
| NM_015559.2(*SETBP1*):c.541-34431_541-31991del | NC_000018.9:g.42495415_42497855del | Het | Hydrocele testis,Torticollis,Abnormality of eye movement,Cyanosis,Seizures,Global developmental delay,Omphalocele,Poor head control,Infantile onset,Focal tonic seizures,Epileptic encephalopathy | Mental retardation type 29, AD (OMIM®:616078) | 2.4 kb deletion in intron 3 |
| NM_001244438.1(*ARG1*):c.330-4A>G | NC_000006.11:g.131902355A>G | Hom | Poor eye contact,Skin rash,Gait ataxia,Broad-based gait,Neurological speech impairment,Aminoaciduria,Abnormal circulating arginine concentration,Childhood onset,Allergy,Abnormal social behavior,Gluten intolerance | Argininemia, AR (OMIM®:207800) | Predicted to affect splicing, very rare, previous lab testing suggested abnormality of arginine metabolism |
| NM_023035.2(*CACNA1A*):c.4878+6T>C | NC_000019.9:g.13363799A>G | Het | Macrocephaly,Low-set ears,Hypotelorism,Intellectual disability,Seizures,Muscular hypotonia,Spasticity,Global developmental delay,Dandy-Walker malformation,Specific learning disability,Hyperreflexia,Joint hypermobility,Obesity,Generalized-onset seizure,Spastic paraparesis,Developmental regression,Myopathy,Skeletal muscle atrophy,Abnormal form of the vertebral bodies,Gowers sign | Early infantile epileptic encephalopathy type 42, AD (OMIM®:617106) | Predicted to affect splicing, novel |

Sub: substitution, Del: deletion, Dup: duplication, Ins: insertion, Hom: Hom, Het: Het, Hem: Hem, Part of Het-Het: Het variants with unknown phase, CH: compound het, proven *trans* phase of alleles, LoF: Loss of function, NDD: neurodevelopmental delay

1: this patient had also Hom VUS in SPTAN1.

**References**

1 Trujillano, D. *et al.* Clinical exome sequencing: results from 2819 samples reflecting 1000 families. *Eur J Hum Genet* **25**, 176-182, doi:10.1038/ejhg.2016.146 (2017).
